# Appraising the value of evidence generation activities: an HIV modelling study

**DOI:** 10.1136/bmjgh-2017-000488

**Published:** 2018-12-07

**Authors:** Beth Woods, Claire Rothery, Sarah-Jane Anderson, Jeffrey W Eaton, Paul Revill, Timothy B Hallett, Karl Claxton

**Affiliations:** 1 Centre for Health Economics, University of York, York, UK; 2 Department of Infectious Disease Epidemiology, Imperial College London, London, UK

**Keywords:** health economics, health policy, health services research, AIDS, HIV

## Abstract

**Introduction:**

The generation of robust evidence has been emphasised as a priority for global health. Evidence generation spans a wide range of activities including clinical trials, surveillance programmes and health system performance measurement. As resources for healthcare and research are limited, the desirability of research expenditure should be assessed on the same basis as other healthcare resources, that is, the health gains from research must be expected to exceed the health opportunity costs imposed as funds are diverted to research rather than service provision.

**Methods:**

We developed a transmission and costing model to examine the impact of generating additional evidence to reduce uncertainties on the evolution of a generalised HIV epidemic in Zambia.

**Results:**

We demonstrate three important points. First, we can quantify the value of additional evidence in terms of the health gain it is expected to generate. Second, we can quantify the health opportunity cost imposed by research expenditure. Third, the value of evidence generation depends on the budgetary policies in place for managing HIV resources under uncertainty. Generating evidence to reduce uncertainty is particularly valuable when decision makers are required to strictly adhere to expenditure plans and when transfers of funds across geographies/programmes are restricted.

**Conclusion:**

Better evidence can lead to health improvements in the same way as direct delivery of healthcare. Quantitative appraisals of evidence generation activities are important and should reflect the impact of improved evidence on population health, evidence generation costs and budgetary policies in place.

**Key questions**

**What is already known?**

- Strategies for HIV investment rely on the availability of relevant evidence but this evidence is subject to uncertainty.
- The value of additional evidence to resolve uncertainties has typically ignored the impact of budgetary policies when managing programme delivery.

**What are the new findings?**

- The conceptual modelling framework shows how to value evidence generation activities to improve decisions made within a vertical HIV disease programme in the face of uncertainty and realistic budgetary policies and compares this to the health opportunity costs of research expenditure.
- The value of evidence generation activities vary across settings that use different budgetary policies, which has important implications for research prioritisation decisions.

**What do the new findings imply?**

- Decision makers charged with prioritising evidence generation activities can maximise population health outcomes in the same way as other healthcare investment by taking account of the health opportunity costs of research expenditure.

## Introduction

HIV/AIDS remains one of the leading causes of death and disability in much of eastern and southern Africa.^1^ Access to effective HIV treatments is increasing^2^ and recent scientific advances have provided interventions that are effective in reducing the risk of HIV acquisition and transmission.^3^ The Joint United Nations Programme on HIV/AIDS (UNAIDS), the Global Fund to Fight AIDS, Tuberculosis and Malaria and the US President’s Emergency Plan for AIDS Relief have emphasised that effective interventions should be prioritised for use in populations in which they deliver most benefit.^4–7^ Survey and mapping methods are now used in many countries to identify areas of high-risk behaviour and high prevalence in order to target interventions to where they offer most benefit.^8^

Cost-effectiveness analysis can support the development of a ‘focused’ strategy for HIV investment,^9 10^ whereby an allocation of resources across risk groups, geographical areas, time^11^ and among competing HIV prevention and treatment strategies is chosen that maximises the health attainable, given the available resources. A focused strategy can lead to greater health impact than a strategy in which the same mix of interventions is used uniformly across a highly heterogeneous epidemic.^10^

The development of a strategy for HIV investments relies on the availability of relevant and reliable evidence. This typically includes evidence relating to the epidemiology and natural history of HIV and how this varies across different populations, the impact of alternative interventions on these processes, the costs of interventions and other healthcare costs. This evidence typically comes from a wide range of sources including clinical trials, surveillance studies, long-term observational studies, costing studies and quality of life surveys. Evidence is often synthesised using decision modelling in order to provide estimates of the costs and outcomes associated with different policy choices over a suitable time frame.^9^

The costs and health gains from local HIV services are inherently uncertain as the underlying evidence base and modelling approach are subject to uncertainty. This uncertainty has two implications.

First, the HIV investment strategy that was expected to maximise population health from available resources may not turn out to be the optimal course of action. This implies that if we had better information when planning the HIV investment strategy, better health outcomes could be achieved from available funding.

The second implication of uncertainty is that budgets allocated to fund specific services in specific populations or geographies may not align with the prevailing situation on the ground. For example, HIV prevalence or costs of care may be higher than expected, so planned levels of population coverage for services may not be attainable with available funds. Where decision makers face hard budget constraints that limit their ability to accommodate cost over-runs, they will require a budgetary policy to be in place in order to manage the cost variances. This implies that if we had better information when planning the HIV investment strategy, the likelihood of deviations from the strategy could be reduced. This represents an important benefit of reducing uncertainty for real-world resource allocation decisions^12^ but has been largely overlooked so far in quantitative appraisals of evidence generation activities.

In this study, we show the health benefits of evidence generation activities when both of these implications of uncertainty are considered. We show the importance of weighing these health benefits against the opportunity costs associated with allocating resources to research rather than service provision. Finally, we show how the value of evidence generation activities depends on the budgetary policy in place.

## Methods

### Overview

A conceptual modelling framework is used to explore the population health implications of investing in evidence generation activities. This model is based on features of a generalised HIV epidemic in sub-Saharan Africa and the policy response to this. We use Zambia as a case study to illustrative the principles of the analyses required and the qualitative implications of alternative courses of action. The model is a simplified representation of HIV epidemiology in Zambia, the HIV prevention and treatment investment opportunities available and the available options for policymakers responding to uncertainty. For these reasons, the results should not be interpreted as providing an accurate quantification of different policy options for Zambia but instead are intended to demonstrate qualitative findings in a relevant setting. A simple transmission model is used to quantify health effects. The aim of the national HIV programme is to allocate a fixed budget to different interventions and across Zambian provinces to maximise population health (expressed in terms of quality-adjusted life years, QALYs). The interventions considered are (1) antiretroviral therapy (ART) in individuals with HIV who present for care due to ill health and who typically have CD4 counts below 350 (henceforth, ‘late ART’); (2) voluntary medical male circumcision in those without HIV infection (‘circumcision’) and (3) ART in individuals with HIV who are identified via outreach testing and who typically have CD4 counts above 350 (‘early ART’). The first stage of national HIV planning involves determining the optimum coverage level of the interventions in each region in order to maximise the expected (average) health outcomes based on currently available information (ie, before the implications of uncertainty unfold). This represents the ‘planned’ HIV investment strategy. This is developed under an additional requirement that late ART is considered an imperative^2^ and decision makers are constrained to scale up provision of late ART until all those with late stage disease receive it. Investment in circumcision and early ART is only pursued if there is sufficient funding to support wide scale provision of late ART.

The second stage involves application of a policy response to uncertainty. Under current information, we initially assume that a *regional policy* operates whereby once the HIV investment strategy has been set and budgets disbursed to regions, the regions must adhere to the intervention budgets allocated. This reflects the fact that recent years have seen a move towards budgets being held locally by decentralised levels of government or non-governmental service providers.

To assess the maximum value of investing in evidence generation activities, we assess the health that could be achieved if current uncertainties were completely eliminated and the values of the model parameters were known that is, the health that could be achieved if we had perfect information. Although in reality further data collection will not resolve all uncertainty, the health generated under perfect information can be compared with the health attained with current information to establish the maximum expected health gains associated with evidence generation activities. This provides the starting point to determine whether additional data collection is worthwhile and whether spending should be devoted to data collection or service delivery.

We then show the impact of the costs of research on the net health benefits of evidence generation activities. These are quantified by comparing the health achieved under current information with the health achieved with perfect information but with a reduced intervention budget (to reflect the resources that must be spent on research). This comparison can be made using different estimates of the resources that must be spent on research. We also estimate the maximum that can be spent on research before it becomes net health reducing that is, the monetary value of research. This can then be used as a benchmark to compare to the costs of the research to determine whether the research is potentially worthwhile.

Finally, we examine how the budgetary policy, by modifying the health attained under current information, impacts on the health benefits of evidence generation. We examine two alternatives to the regional policy response: the national policy and the contingency fund policy, which alongside the regional policy are intended to represent the spectrum of approaches operating in countries in sub-Saharan Africa.

### Model description

A transmission model was used to predict the impact of interventions on the course of the HIV epidemic over a 15-year period 2015–2030. We modelled outcomes in adults aged 15–49 in nine different provinces of Zambia with populations ranging from 400 000 to 1.1 million individuals. Each region represented a locality for which a HIV budget can be administered by a government or non-government organisation. The regions differed in HIV prevalence, which ranged from 5.4% to 18.2%, and the baseline proportion of individuals entering the population who were circumcised, which ranged from 1.6% to 72.6% of men; this resulted in differences across regions in the costs and effects of investments. Individuals entered the population without infection at age 15. A proportion of individuals who do not have HIV faced the risk of infection. Newly infected individuals enter early-stage infection and progress to late-stage infection which worsens their quality of life and prognosis. Full details on study methods are available in online supplementary appendix 1.

10.1136/bmjgh-2017-000488.supp1 Supplementary data

#### Epidemiology and intervention effects

Province-level population data, HIV prevalence and rates of circumcision were obtained from a previous epidemiological model and reflect the parameter values obtained following calibration to a range of key data sources; for further detail, see McGillen *et al*.^13^ Natural history parameters were taken from a range of sources and intervention effects reflect findings from key meta-analyses.^14 15^ Late ART reduces transmission rates, reduces mortality and improves quality of life, circumcision reduces the risk of acquiring infection, while early ART removes the possibility of progression to late-stage disease and reduces transmission. Intervention coverage was assumed constant over time. Regardless of the selected investments, a proportion of the population were assumed to be circumcised independently of the HIV circumcision programme, and provision of ART was scaled up to 80% coverage of individuals with late-stage infections prior to 2015 (considered to reflect universal coverage^2^ among this population).

#### Uncertainty

Uncertainties in HIV prevalence in each region, the impact of circumcision and ART on the risk of transmission and the costs of ART and circumcision delivery were included in the model. Uncertainties were included in the model by assigning a statistical distribution to each uncertain quantity. Different distributions were assigned to prevalence in each region to reflect prior knowledge regarding HIV epidemiology in each region. The same distributions were used across regions for the other uncertain parameters. The uncertainty reflects the fact that the true values of these quantities are unknown. For example, an estimate of HIV prevalence for a particular geographical area may be available from a household survey. Nonetheless, actual prevalence may differ from this estimate as the survey sample may have been small and therefore by chance have contained more or less individuals with HIV than the target population or the sample may not fully match the characteristics of the target population.^8^

#### Economic analysis

Costs per circumcision, per year on ART and of HIV testing were included and reflect comprehensive large scale costing studies conducted in Zambia.^16–20^ Costs were assumed to decrease according to the scale of production but increase at high coverage levels due to the need for outreach activities.^21 22^ QALYs were estimated by allocating disability adjusted life year weights^23 24^ according to the distribution of modelled individuals across different health states. The consequences for population health of generating additional evidence are evaluated at national HIV budgets up to US$23 per capita per annum (pcpa) or ~US$200 per individual with HIV, beyond this point all investment opportunities considered within the model had been exhausted.

#### Health outcomes under different informational and budgetary scenarios

**Figure 1:**
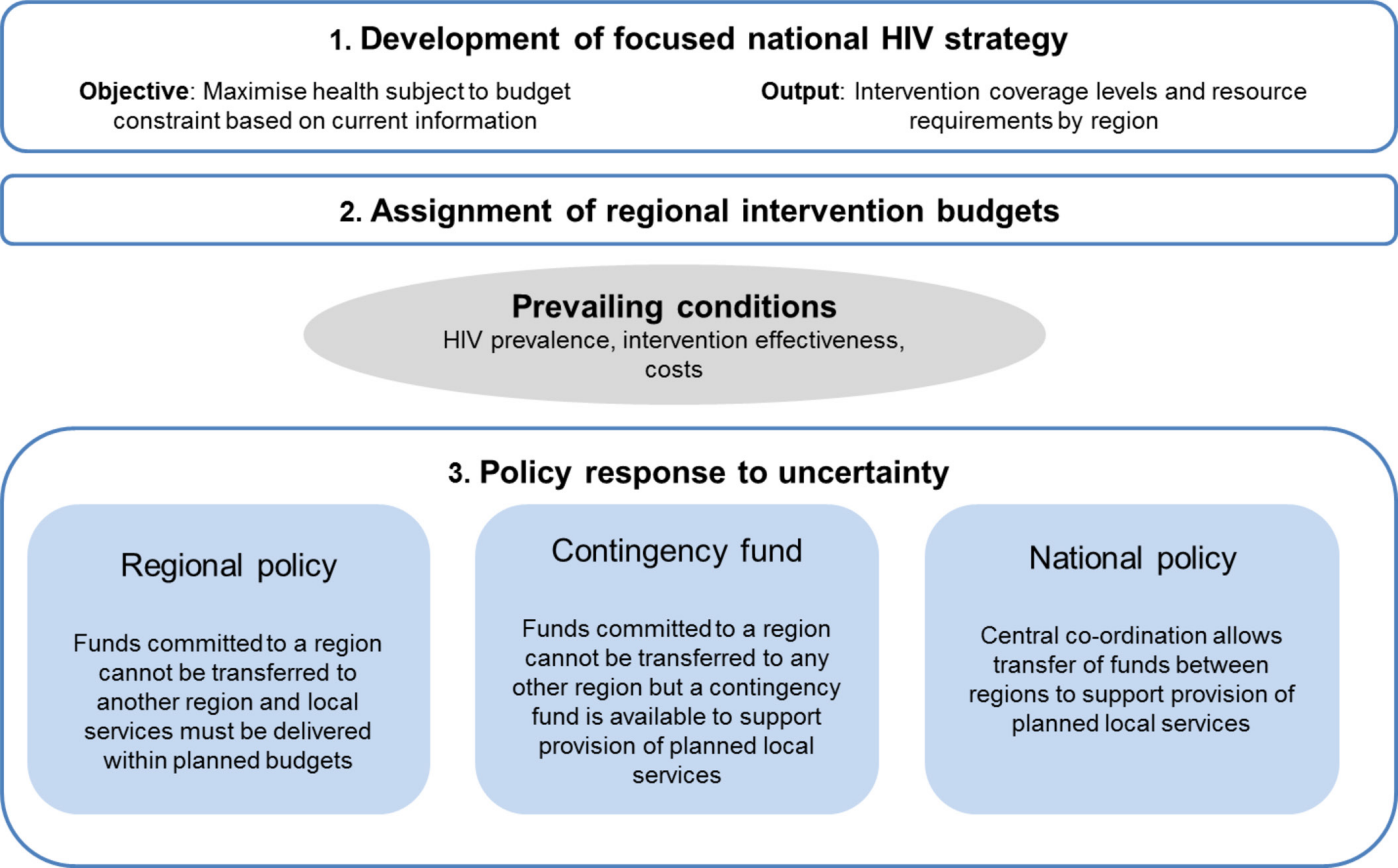
Policy response to uncertainty under current information.

All policies involved a first stage in which the planned HIV investment strategy was developed to maximise health given the available resources and based on currently available data (figure 1). This provided a plan for the coverage levels for each intervention in each region and how resources should be allocated between regions.

Under current information and a *regional policy,* decision makers spent what they initially planned on each intervention in each region. They did not reallocate resources across interventions or have means by which to extend their total regional funds beyond those allocated. This ensured regional spending did not exceed the budget; however, it did not guarantee that regions were able to achieve the coverage planned. For example, if HIV prevalence was higher than expected, available funds may not be sufficient to deliver on a commitment to universal late ART coverage. If the funding for an intervention within a region exceeded that required to deliver the planned coverage level, then intervention coverage was scaled up to the point at which the allocated funds were exhausted unless this exceeded the maximum feasible coverage level, in which case coverage was capped at this maximum and any further funds that were allocated to that intervention were used ineffectively. This was considered to be a close model of the challenges faced when managing funds under uncertainty. This simulates the situation whereby once funds are committed to a region and intervention programme, it is not possible for those funds to be transferred to another region or intervention.

Under *perfect information* the values taken by the model parameters are known and the HIV investment strategy can therefore be revised for each realisation of uncertainty. This ensures that the investment strategy delivers the maximum health from available resources, and removes the possibility of cost variances as budgets are always set correctly. The difference between the health achieved with perfect information (parameter values known, no uncertainty) and the health achieved under current information (parameter values subject to uncertainty with a regional policy response to the uncertainty) represents the expected maximum health benefits of evidence generation activities (ie, the expected value of perfect information, EVPI).^25^

Under current information and a *national budgetary policy* total spending across regions was required to remain within the national HIV budget but funds could be transferred between regions to support implementation of the planned investment strategy. If the total cost of the investment strategy exceeded the national HIV budget then expenditure was scaled down by the same proportion across interventions and regions. If total costs fell below the national budget spending was scaled up until the budget was exhausted or the maximum coverage reached. This simulates the situation where there is central co-ordination that allows the transfer of funds between regions to support the provision of planned local investments.

Under the *contingency fund* policy a proportion of the total HIV budget was set aside prior to development of the investment strategy (a 5% contingency fund was used as this generated the most health in this example, see online supplementary appendix 1) to support provision of planned local investments when uncertainty unfolds. Decision makers planned to spend the total HIV budget less the amount dedicated to the contingency fund. Any intervention service experiencing costs that exceeded their allocated budget could use the contingency fund to preserve their planned coverage. If the contingency fund was insufficient to pay for all claims, each claim was reduced by the same proportion. Any contingency funds that were not required were assumed to be used ineffectively. This simulates a similar situation to the regional policy but whereby there is also a centrally co-ordinated fund to support provision of local investments.

## Results

### HIV investment strategy based on current information

**Figure 2:**
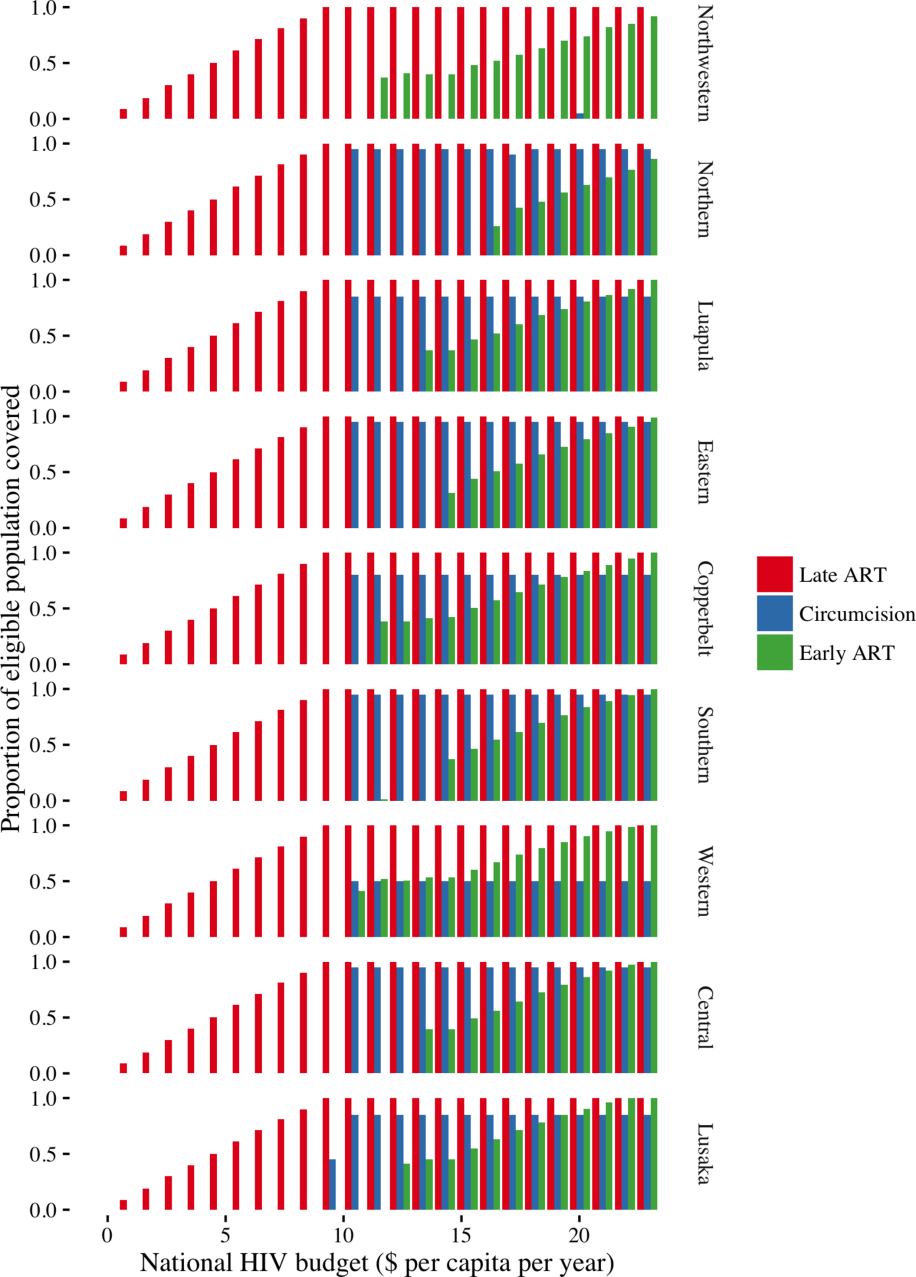
Intervention coverage by region and budget (regions ordered according to HIV prevalence, from lowest to highest).

The optimal coverage level for the interventions based on maximising expected health outcomes using currently available information varies by region and national HIV budget (figure 2). At low budgets, investments focus on late ART as universal access is a priority. As budgets increase, investment shifts to circumcision as this is the most cost-effective intervention. At higher budgets, investments in early ART start. Investments in early ART are initially focused on regions with a high prevalence and/or high baseline rate of circumcision, as observation of these features implies that a higher proportion of susceptible individuals are at risk of contracting HIV, reflecting difference in sexual behaviours across regions. The resulting higher transmission risk in these regions make the preventative effects of early ART particularly valuable. For example, in the Northwestern province, the proportion of the population entering the circumcised group at model entry (baseline) is much higher than any of the other regions (36.3% vs <5% in most other regions)—see online supplementary table S1. This means that to produce the observed prevalence level in 2013 for this region, the proportion of susceptible individuals at risk of contracting HIV is inferred to be relatively high. This makes early ART which is very effective at preventing transmission a better buy than circumcision in this region.

### Maximum population health benefits from evidence generation activities

**Figure 3:**
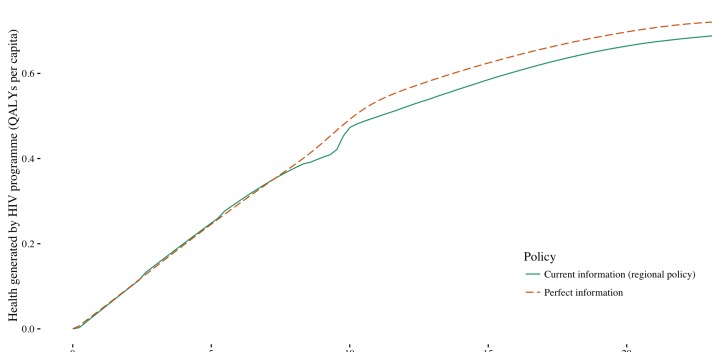
Health generated under current information (regional policy) and perfect information at a range of national HIV budgets.

We assessed the maximum expected value of improved information if this information were to become available before budgets are set and coverage decisions made. This maximum value can be estimated by comparing the health generated under perfect information to the health generated under current information. Perfect information improves health (figure 3) since it ensures that planned programmes are fully tailored to the prevailing conditions and removes the need to modify planned programmes in response to uncertainty. The health gain achieved at a specific budget can be estimated as the vertical difference between the current information and perfect information curves. At lower budgets, there is little difference between perfect information and current information. This is because while universal access is a priority, the decision maker has little opportunity to respond to prevailing conditions as they are constrained to only invest in late ART and to provide even coverage across regions. (At some budgets, it is actually possible for the current information policy to perform better than the perfect information policy. This occurs because we do not ‘constrain’ the remedial actions, eg, we do not require equal coverage across regions.)

### Reflecting the opportunity costs of evidence generation expenditures

**Figure 4:**
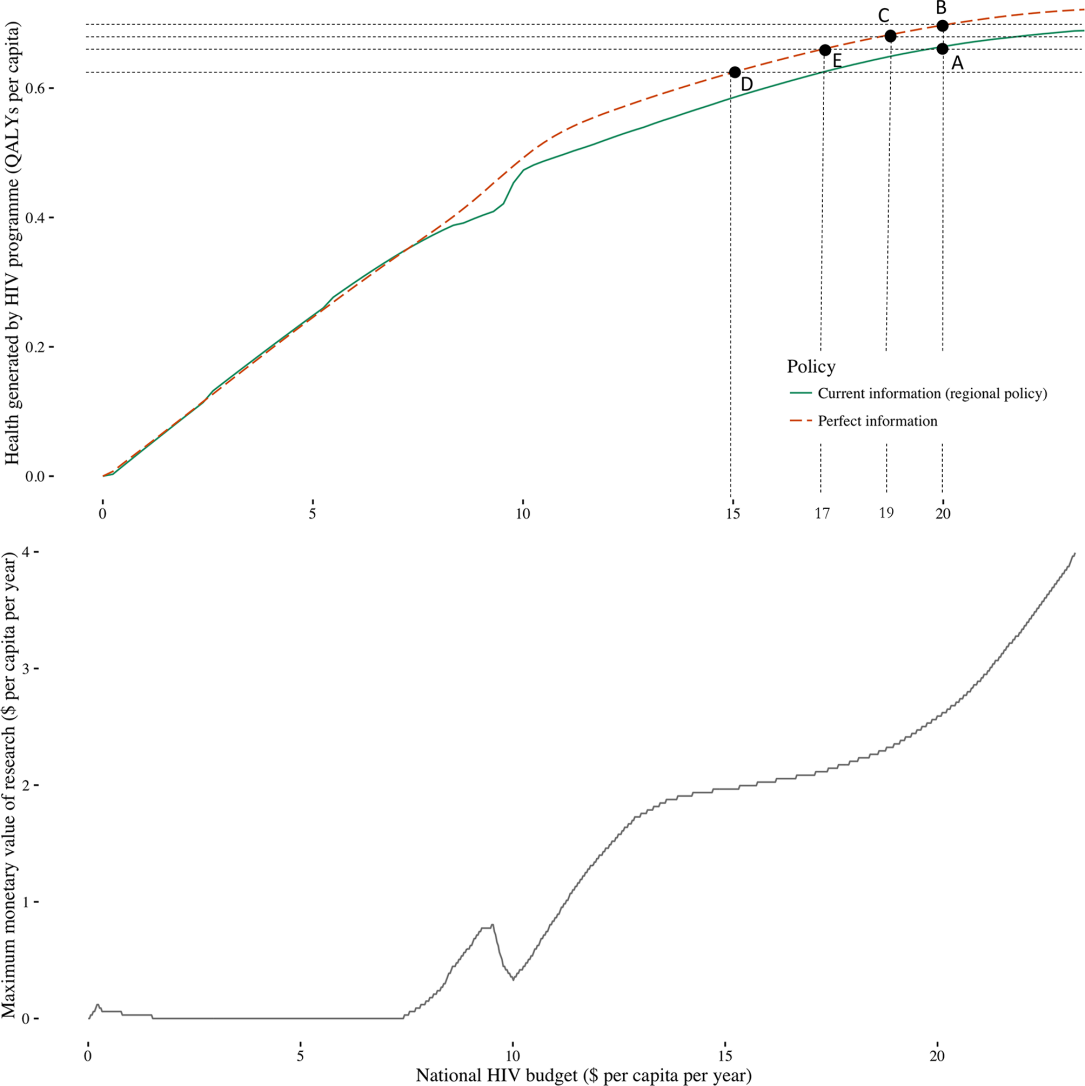
(A) Comparing the health gains of research to the health opportunity costs of research funding and (B) the maximum that could be spent on research before reducing population health.

Improved information provides a means through which to improve population health (figure 3). However, the difference between the health attained with perfect information and that attained with current information does not account for the impact on health of spending funds on research which could otherwise have been used to fund services. For example, at a HIV budget of US$20 pcpa, the health benefits of research are shown in figure 4A as the vertical distance between points A and B. If the research costs US$1 pcpa, the remaining intervention budget is US$19 pcpa and the net health gains of evidence generation, taking into account research costs, are shown by the vertical distance between A and C.

If the research costs US$5 pcpa, the remaining budget would be US$15 pcpa. In this instance, the intervention budget left over after research is sufficiently low that the health benefits of research are outweighed by the costs imposed and there is a net health loss which is the vertical distance between A and D.

We are able to identify the point at which the health benefits of research are exactly the same as the costs so we would be indifferent as to whether the research went ahead or not, this is shown by point E. Point E occurs at a budget of US$17 pcpa when US$3 pcpa has been spent on research. This US$3 represents the maximum that could be spent on research before the net health effects become negative. This maximum monetary value of research can be estimated for any budget (figure 4B). Although the health gains associated with perfect information level off at higher budgets (figure 4A), the monetary value of research continues to rise (figure 4B). This reflects the fact that the opportunity cost of healthcare funds are lower at higher budgets (there are fewer remaining high value investments to make) so we are willing to give up more budget to achieve similar health gains from research. The true monetary value of a specific evidence generation programme will be lower because uncertainty can only be reduced rather than eliminated (see the Discussion section). Therefore, the amount that can be spent on research while still generating population health benefits will also be lower.

### The impact of budgetary policies on the value of evidence generation

**Figure 5:**
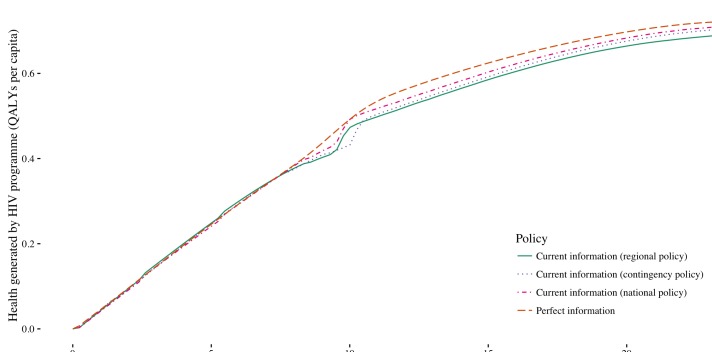
Health generated under current information with the regional, contingency and national budgetary policies and perfect information at a range of national HIV budgets.

Under current information, a national policy response to uncertainty improves population health compared with a regional policy (figure 5) as funds can be moved between regions and interventions to support planned intervention coverage levels as uncertainty is realised. Cost over-runs at planned coverage levels can be met by releasing funds from where there are cost under-runs at planned coverage levels, thus reducing the likelihood of substantial deviations from the original investment strategy. When the contingency fund policy operates, the planned investment strategy is more likely to be delivered, but there is also a higher chance that the investment strategy is conservative and contingency funds are not used effectively. At some budgets, the contingency fund performs worse than the regional policy. In these circumstances, the use of the contingency fund to support the delivery of late ART is not sufficient to offset the potential loss of health outcomes from unspent contingency funds. At higher budgets, the contingency fund generates more health than the regional policy as although planned investment in early ART is lower, there is an increased likelihood that more cost-effective interventions—circumcision and late ART—are provided as planned.

As the national and contingency fund policies generally improve the population health attained under current information, they reduce the gains in population health attainable via perfect information (figure 5). The flexibility afforded by these policies reduces the costs of uncertainty, and therefore the benefits achievable by eliminating uncertainty. This indicates that the value of evidence generation activities—and therefore the amount that should be invested in funding them—depends on the budgetary policy in place.

## Discussion

This work provides practical insights into the population health consequences of uncertainties in the evidence supporting healthcare decisions and the policy levers available to mitigate these. We show that the collection of further information to reduce uncertainty at the point at which the HIV investment strategy is being developed and budgets assigned has the potential to improve population health. Indeed, guidance issued by the WHO and UNAIDS emphasises detailed collection of epidemiological data as an essential first step for informing the strategic direction of resources.^26^ We show that robust data can both improve the design of the investment strategy and the likelihood that any planned investment strategy will actually be implemented. However, it is insufficient to quantify the potential health gains associated with improved data. Evidence is costly to generate, so the health opportunity costs imposed by investment in research rather than service delivery must be calculated to determine if research investments will result in a net improvement to population health. Finally, we show that the health benefits of research will depend on the budgetary policy in place. The value of evidence generation is higher when restrictive budgetary policies are in place as these policies reduce the population health attained under current information. This study used a case study in HIV but the findings are general to all situations in which resources are allocated to healthcare programmes and there is uncertainty relating to the costs and effects of those programmes. Although other studies have demonstrated the utility of methods for evaluating the value of evidence generation activities in the global health setting,^27^ these studies have not attempted to incorporate decision makers responses to cost under-runs or over-runs, which can have important implications for how evidence generation activities are valued. The methods presented show how quantitative appraisal of evidence generation activities can be conducted. These methods can be used to support more accountable decision-making among organisations charged with allocating limited funds to primary data collection efforts (eg, trials, surveys) and secondary data analysis.

Our results also suggest that in the face of uncertainty, the budgetary policy may represent an important policy lever through which to improve population health. For example, health gains may be achievable by moving from a regional to a national policy, though this would require a wider appraisal of the various costs and benefits of operating a more centralised system. The budgetary policies presented were intended to represent the range of situations operating within low-income countries. For example, the regional policy reflects a decentralised system in which transfers of funds do not occur or are difficult to effect (eg, in Kenya, HIV and other healthcare resources are now allocated and controlled at county level),^28^ the national policy reflects systems with more central coordination where reallocations are feasible (eg, via budget virements as used in Malawi) and the contingency policy reflects a situation that may operate informally or formally in some systems (eg, ‘unallocated reserves’ as in Sierra Leone). These scenarios reflect our understanding of policy responses to uncertainty established via discussions with individuals with experience of national-level HIV resource allocation planning and delivery and individuals with research interests in public financial management. The policy scenarios include assumptions about how decision makers might respond to having more or less funds than required to meet their original coverage goals. If these assumptions do not hold then the results will change. For example, if decision makers with excess funds transfer these funds to productive uses within other health-improving intervention programmes, then more health could be generated under current information, which reduces the benefits of evidence generation activities. A similar line of argument applies to the assumption that unused contingency funds are used ineffectively. If this is not the case and they are used to fund other health-generating activities then this would increase the health generated under current information and reduce the value of further evidence generation activities. More formal qualitative and quantitative research is warranted to better characterise how resource allocation decisions respond to unfolding uncertainties.

In this work, we estimated the expected maximum value that could be derived from removing all uncertainty relating to a specific set of policy questions. Estimating the value of removing all uncertainty provides the starting point to determine whether additional data collection efforts are worthwhile. If the cost of potential new research activities exceeds the value of removing all uncertainty, then further evidence generation is unlikely to be worthwhile. However, if this is not the case, the next step would be to quantify the value of specific data collection activities which would be expected to reduce some uncertainties rather than completely eliminate all uncertainty. Evaluations of specific evidence generation activities should reflect the degree to which new data will reduce uncertainties, the value of resolving these uncertainties, the timeliness with which data can be collected and reported, whether commissioning research would have any implications for the HIV investment strategy today (eg, it may not be possible to adopt an intervention while randomising individuals within a trial to that intervention), uncertain future changes in prices/technologies/the evidence base, decision makers response to new information and to cost over-runs and under-runs, any response by other actors to unfolding events (eg, individuals seeking care in regions with better resource availability), the full set of constraints in place (eg, constraints on feasible resource allocations, constraints on the availability of key inputs such as healthcare workers) and the costs and timing of collecting data (eg, some data collection activities such as epidemiological surveys are generally conducted at regular intervals to provide up to date information).^29 30^ For some types of evidence, value may extend beyond the policy questions considered, for example, evidence on the relative effects of interventions may generalise across jurisdictions.^31^ In such cases, some estimate of the scale of these benefits will be required. Given the complexity and futility of attempting to formally model all of these considerations, there is a need for pragmatic guidance on conducting quantitative assessments of evidence generation activities to inform real-time policy decisions.

## Conclusion

The development of a strategy for HIV investment, and in fact any investment in healthcare, relies on the availability of relevant and reliable evidence. This evidence and the process by which it is synthesised to support decision making are inherently subject to some level of uncertainty. We show that the collection of further information to reduce these uncertainties at the point at which an investment strategy is being developed and budgets assigned has the potential to improve population health. Better evidence can both improve the design of the investment strategy and the likelihood that any planned investment strategy will actually be implemented, thus leading to health improvements in the same way as direct delivery of healthcare. However, as resources for healthcare and research are limited, the desirability of research expenditures should be assessed on the same basis as other healthcare resources, that is, the health gains from research must be expected to exceed the health opportunity costs imposed as funds are diverted to research rather than service provision. Quantitative appraisals of evidence generation activities offer the opportunity to support a more accountable process for allocating health-related funding to evidence generation activities, and further pragmatic guidance on conducting these types of assessments in HIV and global health more generally is required.
