# Supplementary material for: Appraising the value of evidence generation activities: an HIV modelling study
Source: BMJ Glob Health. 2018 Dec 7;3(6):e000488. doi: 10.1136/bmjgh-2017-000488 (PMC6304099; doi:10.1136/bmjgh-2017-000488)
Supplement: Supplementary data [file bmjgh-2017-000488supp001.pdf]

Supplementary material

1 Detailed description of transmission model 2

2 Detailed description of resource allocation model 11

3 Implementation of simulation modelling 17

4 Software 18

5 Additional results: size of the contingency fund 17

6 Supplementary material references 25

1 Detailed description of transmission model

A simple dynamic transmission model is used to model HIV transmission independently in nine regions within Zambia. The model is run over the period 1980-2030. The HIV interventions considered are late antiretroviral therapy (ART), voluntary male circumcision and early ART. Late ART describes the provision of ART for those who present for care, usually due to ill health, and who typically have CD4 counts below 350. Early ART describes the provision of ART to individuals who are identified via active outreach testing and who typically have CD4 counts above 350. Early and late ART reduce the probability of onward transmission of infection as well as resulting in direct health benefits to the individuals receiving treatment: early ART removes the risk of progression to late stage infection and late ART improves quality of life and reduces the risk of HIV-related death. Circumcision reduces the risk of men acquiring HIV infection.

The model structure is shown in Figure S1. States S1-S4 denote individuals susceptible to infection: S1 denotes circumcised men not at risk of infection, S2 circumcised men at risk of infection, S3 non-circumcised individuals at risk of infection and S4 non-circumcised individuals who are not at risk of infection. I1 denotes individuals in the earlier infection state who have CD4 counts above 350 and I2 individuals in the second later infection state who have CD4 counts below 350. EART and LART denote individuals receiving early and late ART respectively. Sex and variation in propensity for risky sexual behaviour are not explicitly incorporated in the model.

Individuals enter the modelled population at age 15. The number of individuals entering the population is denoted *b*. The proportion of individuals entering the population who are not at risk of infection (τ), and the proportion who are circumcised (c) determine how individuals are distributed across states S1 to S4 at model entry. The model is calibrated to a set of region-specific estimates of HIV prevalence for the year 2013. This is achieved by identifying the value of τ that minimises the sum of squared differences between the estimates of HIV prevalence and the model prediction of HIV prevalence for each region. This allows differences in sexual behaviours across regions to be reflected in the model. λ_C and λ_{NC} describe the force of infection in circumcised and uncircumcised individuals, respectively. σ describes the rate of progression from infection state I1 to I2. Individuals face different mortality rates depending on their health state. All individuals face a risk of leaving the sexually active population of μ_G, individuals with early stage infection face an additional risk of death (at rate μ_E); as do individuals receiving late ART (μ_L) and those who are untreated and in the more severe infection state (μ_I). The birth rate is (μ_G +g) where g represents the population growth rate. ϕ_E and ϕ_L denote the proportion of those eligible for early and late ART who receive treatment, respectively. In addition the dashed arrows indicate one-off transitions possible at discrete points in the model. These are used to reflect immediate scale up of interventions as discussed below.

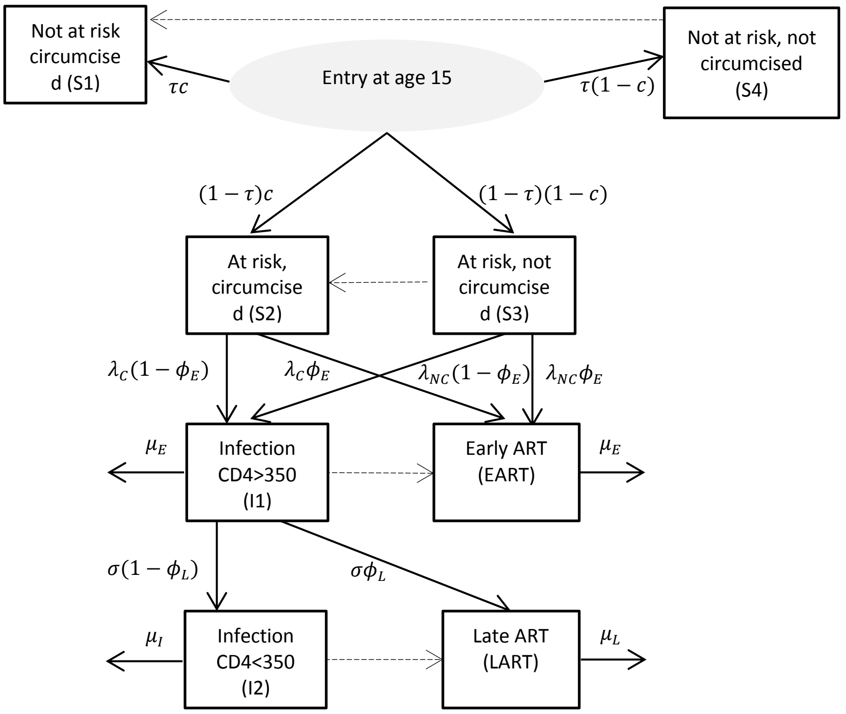

Figure S1: Schematic diagram of transmission model. Squares describe health states. Arrows show possible transitions and arrow labels indicate transition rates. Individuals can exit the sexually active population from all states at rate μ_G (not shown).

The model is described by the following differential equations:

$$\frac{dS1}{dt} = b\tau c - \mu_G S1$$

$$\frac{dS2}{dt} = b(1-\tau)c - \left(\lambda_C + \mu_G\right)S2$$

$$\frac{dS3}{dt} = b(1-\tau)(1-c) - \left(\lambda_{NC} + \mu_G\right)S3$$

$$\frac{dS4}{dt} = b\tau(1-c) - \mu_G S4$$

$$\frac{dI1}{dt} = (1-\phi_E)(\lambda_C S2 + \lambda_{NC}S3) - (\sigma + \mu_G + \mu_E)I1$$

$$\frac{dI2}{dt} = (1-\phi_L)\sigma I1 - \left(\mu_G + \mu_I\right)I2$$

$$\frac{dEART}{dt} = \phi_E(\lambda_C S2 + \lambda_{NC}S3) - \left(\mu_G + \mu_E\right)EART$$

$$\frac{dLART}{dt} = \phi_L \sigma I1 - (\mu_G + \mu_L) LART$$

The force of infection is described by the following equations:

$$\lambda_C = (1 - \varepsilon_C)\beta \frac{(I1 + I2 + (1 - \varepsilon_A)(EART + LART))}{(S1 + S2 + S3 + S4 + I1 + I2 + EART + LART)}$$

$$\lambda_{NC} = \beta \frac{(I1 + I2 + (1 - \varepsilon_A)(EART + LART))}{(S1 + S2 + S3 + S4 + I1 + I2 + EART + LART)}$$

where ε_C is the reduction in the risk of acquisition of HIV in individuals who have been circumcised, β is the rate of transmission and ε_A is the reduction in the rate of transmission from individuals receiving early or late ART.

b is determined by the population 15 years prior to the current time period to avoid the choice of interventions impacting on the number of individuals entering the population.

The model focuses on intervention choices faced in 2015 and the impact of these choices over the period 2015-2030. To accurately reflect the evolution of the epidemic we also include intervention use prior to 2015. In 2006 there is an immediate scale up of ART treatment for those living with late stage infections (shown by the dashed arrow from I2 to LART) and in 2006-2015 there is a gradual scale up in the proportion of those progressing to late stage infection who receive ART. There is also a region-specific baseline level of circumcision (c) throughout 1980-2030. No provision of early ART is included prior to 2015.

During the intervention period (2015-2030) a proportion (ϕ_L) of those progressing from early to late stage infection receive ART. This proportion can range from 0 to 80%, as up to 80% of individuals with late stage infection can be identified and are willing to undergo treatment.(1) A proportion (ϕ_E) of newly infected individuals and individuals infected prior to 2015 receive early ART (the latter shown by the dashed arrow from I1 to EART). This proportion can range from 0 to 73% as up to 73% of individuals with early stage infection can be identified and are willing to undergo treatment.(2) A target circumcision rate is set and at the beginning of the intervention period individuals are moved from state S4 to S1 and from S3 to S2 to meet this target, as shown by the dashed arrows. The number of people who make the transition from S3 to S2 (ρ_{ar}) and S4 to S1 (ρ_{nar}) are:

$$\rho_{ar} = (c + \phi_C) \cdot (S2 + S3) - S2$$

$$\rho_{nar} = (c + \phi_C) \cdot (S1 + S4) - S1$$

where $\phi_{C,}$ is the desired increase in the proportion of individuals circumcised. We assume that it is possible for up to 40% of all individuals to be circumcised (i.e. approximately 80% of all males corresponding to half of the modelled population). The values taken by $\phi_{C,}$ therefore depend on the region-specific baseline level of circumcision, c. For example, if the baseline level of circumcision is 25% ϕ_C can take values of between 0% and 15%.

Any combination of these coverage levels for circumcision, early ART and late ART can be selected by choosing the corresponding values for ϕ_L, ϕ_E and ϕ_C. The way in which coverage levels are selected is determined by the resource allocation component of the model, which is discussed in the next section.

Each health state is associated with a different health related quality of life weight to reflect differences in the morbidity of individuals in the model. Costs are incurred for each circumcision, each year of ART and each HIV test required. Early ART involves active outreach to identify HIV positive individuals in the community, and therefore incurs a testing cost. The number of tests is calculated by multiplying the numbers of individuals initiating early ART by the number of individuals who must be tested to identify each HIV positive individual.

Costs depend upon the scale of intervention provision due to both the need to employ additional outreach measures, such as travel vouchers, to achieve higher coverage levels, and economies of scale. We follow the approach taken by Meyer-Rath et al. in order to quantify these effects.(3) If total coverage exceeds 40% costs

increase by a percentage (v) to reflect outreach activities. This percentage increase depends upon the coverage level and elasticity of demand (e):

$$v = \frac{\ln(\frac{coverage}{1-coverage}) - \ln(\frac{0.40}{1-0.40})}{e}$$

Outreach costs are calculated independently for early and late ART as these interventions relate to distinct populations of individuals. Increasing returns to scale are modelled for a proportion (α) of total costs. These costs respond to scale of provision according to a scale elasticity of γ, where γ=0.5 implies that a 10% increase in the number of individuals receiving an intervention results in a 5% increase in costs. The returns to scale associated with ART depend on the total number of individuals receiving late or early ART.

The parameter values informing the transmission model are shown in Table S1.

Uncertainty in the following key parameters is reflected in the model: prevalence in the year 2013 in each region; the effect of ART and circumcision on transmission ($\varepsilon_A, \varepsilon_C$) and the cost of circumcision and ART therapy. All other parameters within the model are held fixed. Parameter uncertainty is propagated through the model via Monte Carlo simulation, this involved randomly sampling from a distribution assigned to each uncertain parameter and running the model for each set of sampled parameters, 600 simulations were used. Each simulation represents one 'realisation' of uncertainty or 'state of the world' that could occur. Each parameter is sampled independently across regions to reflect differences in epidemiology and ability to deliver care effectively and efficiently.

The model provides estimates of total costs and total health outcomes, expressed as quality adjusted life years (QALYs), for each region.

Table S1: Summary of input parameters applied in the transmission model

Variables	Value	Source
**Population characteristics**		
Population growth rate, *g*	3% per annum	Representative of sub-Saharan African setting (4)
**Time**		
Model time horizon	50 year	Assumption
Time step used in solving ordinary differential equations	0.05 (i.e. 20 steps per annum)	Assumption
Intervention implemented	Year 35 of model (2015)	Assumption
**Initial conditions for transmission model**		
Initial population size in each province (aged 15-49 years)		
Central	680,160	Data taken from epidemiological model of sub-Saharan Africa and reflect calibration to a range of key data sources.(5)
Copperbelt	1,023,700	
Eastern	827,260	
Luapula	514,680	
Lusaka	1,140,500	
Northern	572,540	
Northwestern	376,360	
Southern	827,480	
Western	470,660	
Proportion of population initially HIV infected in 1980	0.5%	Assumption
Proportion of population entering circumcised group at model entry, c, by province		
Central	2.4%	Data taken from epidemiological model of sub-Saharan Africa and reflect
Copperbelt	7.7%	
Eastern	0.8%	

Variables	Value	Source
Luapula	4.6%	calibration to a range of key data sources.(5)
Lusaka	4.8%	
Northern	2.4%	
Northwestern	36.3%	
Southern	1.2%	
Western	18.7%	
Proportion of prevalent late stage infections receiving ART 2006	80%	Reflects typical late ART scale up to universal coverage.
Proportion of incident late stage infections receiving ART 2006,07,08,09,10,11,12-15	0%, 13%, 27%, 40%, 53%, 67%, 80%	
**Epidemiological conditions**		
HIV prevalence in 2013 amongst individuals aged 15-49 years in each region (mean, 95% confidence interval, beta distribution used to represent uncertainty)		Data taken from epidemiological model of sub-Saharan Africa and reflect calibration to a range of key data sources.(5)
Central	14.7 (8.9, 20.4)	
Copperbelt	11.8 (7.2, 16.4)	UNAIDS present "uncertainty bounds" around estimates of HIV prevalence that aim to reflect the quantity of data, it's relevance to the population of interest and the degree to which assumptions were required to generate estimates. If these uncertainty bounds were taken to represent 95% confidence intervals they would imply standard errors values that were approximately 3-6% of the mean values. As in this model prevalence is the only source of uncertainty relating to the HIV epidemic that we reflect we assume that the standard errors are approximately 20% of the mean values.
Eastern	10.5 (6.4, 14.6)	
Luapula	10 (6.1, 13.9)	
Lusaka	18.2 (11, 25.3)	
Northern	5.9 (3.6, 8.2)	
Northwestern	5.4 (3.3, 7.5)	
Southern	12.7 (7.7, 17.7)	
Western	13.3 (8.1, 18.6)	
**Rate of transmission and progression**		
Rate of transmission in sexually active population, β	0.60	As there is no risk structure in the model a high transmission rate is used to reflect conditions which would result in high HIV prevalence: high partner change rates, high numbers of sex acts and low condom use.
Rate of progression from early to late stage infection, σ	0.11	Time from early to late infection of ~9 years (6)
**Intervention parameters**		
Target increase in proportion circumcised, ϕ_C	0-39% depending on region as regions have different baseline levels of circumcision	The maximum proportion of men circumcised (39%) corresponds to a 78% increase in circumcision coverage in men (as we assume men account for half of the modelled population).
Proportion eligible receiving late ART, ϕ_L	0-80%	80% generally considered to represent universal coverage (1).
In the presence of intensified outreach for ART, the proportion eligible to receive ART prior to late stage infection , ϕ_E	0-73%	73% used to reflect maximum expected based on current targets(2)
Reduction in risk of acquiring HIV infection in those circumcised, ε_c (mean (95% CI), beta distribution used to reflect current uncertainty)	0.54 (0.38, 0.66)	Meta-analysis of key randomised controlled trials.(7)
Reduction in rate of transmission for early or late ART treatment, ε_A (mean (95% CI), distribution used to reflect current uncertainty)	0.86 (0.66, 0.94)	Reflects meta-analysis of comparative studies.(8)
**Mortality rates**		

Variables	Value	Source
Rate of exit from sexually active population, μ_G	0.029	Assume in sexually active population for ~35 years
Rate of death in individuals with early stage infection, μ_E	0.029	Assumption
Rate of death in individuals receiving late ART treatment, μ_L	0.050	~20 years life expectancy on ART(9)
Rate of death in untreated late stage HIV infected individuals, μ_I	0.173	~5.8 years life expectancy (6)
**Health-related quality of life**		
QALY weight for all susceptible individuals (S1, S2, S3, S4)	1	
QALY weight for I1 infected state	0.947	1-general population-derived DALY weights {Anderson, 2014 #1;Eaton, 2013 #20}
QALY weight for I2 infected state	0.453	
QALY weight for EART	0.947	
QALY weight for LART	0.947	
**Unit costs, US dollars (2015)**		
HIV test per individual tested	$13	Data from the Optimising the Response in Prevention: HIV Efficiency in Africa (ORPHEA) project indicate an average cost for Zambia of $13 for HIV testing and counselling for 2013 (10). This study used a representative sample of geographies; facility ownership/management (e.g. government vs. non-government) and level of service provision (e.g. hospitals and primary care) and collected data from databases, records and reports and by time motion study.
Circumcision intervention per circumcision (mean (95% confidence interval), gamma distribution used to reflect current uncertainty	$52 ($32,$72)	Martin et al.(11) report a cost of $47 for 2007, based on detailed questionnaires filled in by interview with clinical and administrative staff at health facilities in Zambia and by various officers in the ministry of Health. Initial data from Zambia reported by the ORPHEA project indicate a similar value of $51 for 2013 and this estimate is used in the model.(12) Estimates of uncertainty were not reported and are therefore based on an assessment by the study team.
Early and late ART intervention per individual per annum (mean (95% confidence interval), gamma distribution used to reflect current uncertainty	$273 ($167, $380) gamma	Data from the Multi-Country Analysis of Treatment Costs (MATCH) study are considered to be the most representative data to date with stratified sampling according to facility size or type (small clinic vs. large hospital), location (rural vs. urban), and funding, and collation of data from a range of facility documentation.(13) The study reported mean costs per person per year of $251 for 2010. Although this cost may have dropped due to ARV prescribing and price changes, other changes such as viral load monitoring have had a counteracting effects and recent policy briefings from the

Variables	Value	Source
		National HIV/AIDS/STI/TB council support a cost estimate of $265/$270 for 2016-2020 (in 2015 $).(14) Estimates of uncertainty were not reported and are therefore based on an assessment by the study team.
**Outreach and production function parameters**		
Elasticity of demand, e (ART and circumcision)	10	Reflects outputs from Meyer-Rath et al. (3), no data for circumcision so assumed equal to ART(15)
Proportion of costs that are scale dependent, α (ART and circumcision)	0.33	Previous studies in ART (3) and circumcision(16)
Scale elasticity, γ (ART)	0.80	Meyer-Rath (3)
Scale elasticity, γ (circumcision)	0.50	Bollinger et al. (16)

The model is calibrated to a set of region-specific estimates of HIV prevalence for the year 2013. These were sourced from an epidemiological model to reduce HIV incidence across sub-Saharan Africa (McGillen et al, 2016) {McGillen, 2016 #60}. The HIV prevalence estimates of McGillen et al (2016) reflected calibration to a range of key data sources, including – but not limited to – Demographic and Health Survey (DHS) data {Central Statistical Office (CSO) [Zambia], 2015 #70}. The difference in HIV prevalence (%) in the year 2013 among individuals aged 15-49 years in each region between the two data sources, McGillen and DHS Survey for 2013-2014 is shown in Figure S2(a). The standard error or confidence intervals for the DHS estimates were not reported by region, making it difficult to assess whether the estimates overlap or not. Any differences noted between the two sources will be due to the structure of the model and assumptions. Importantly, the model is not intended to accurately reflect HIV prevalence and epidemiology in Zambia, or to inform decision making in Zambia. Instead the model has a set of geographic units with a range of epidemiological conditions and magnitudes of epidemic that is consistent with several real countries, including Zambia

The uncertainty around the prevalence estimates included in the model in each region is based on a beta distribution as shown in Figure S2(b).

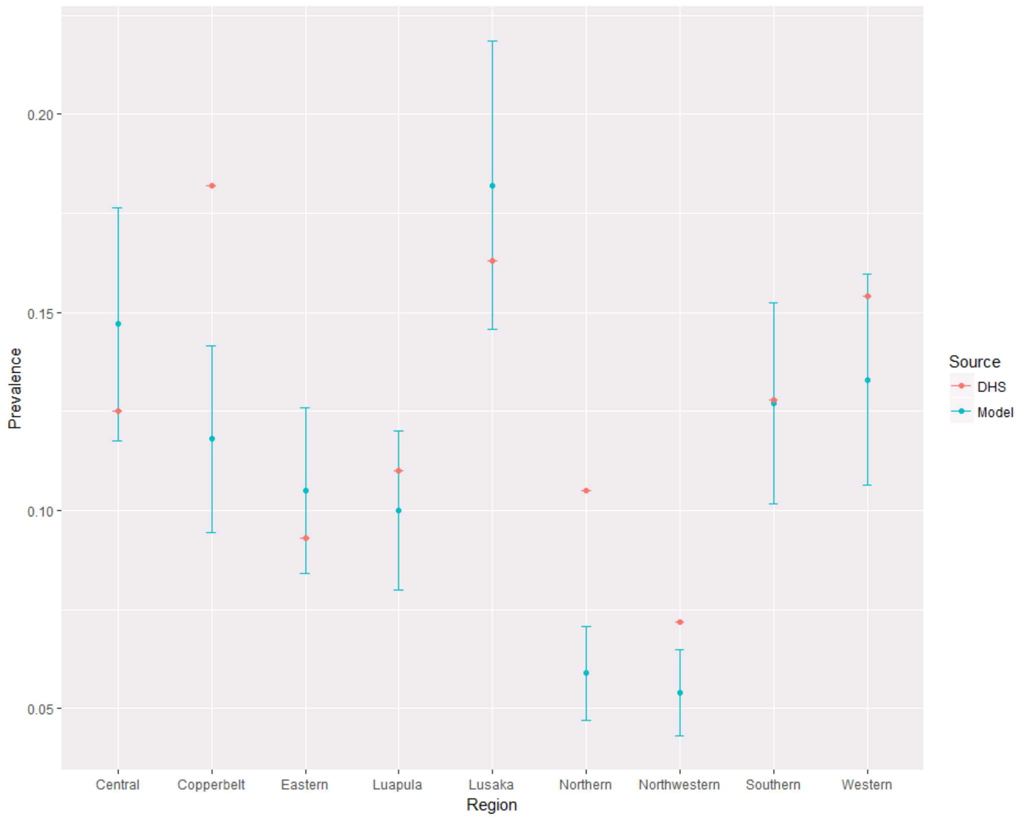

Figure S2(a): HIV prevalence estimates (%) by region among individuals aged 15-49 years

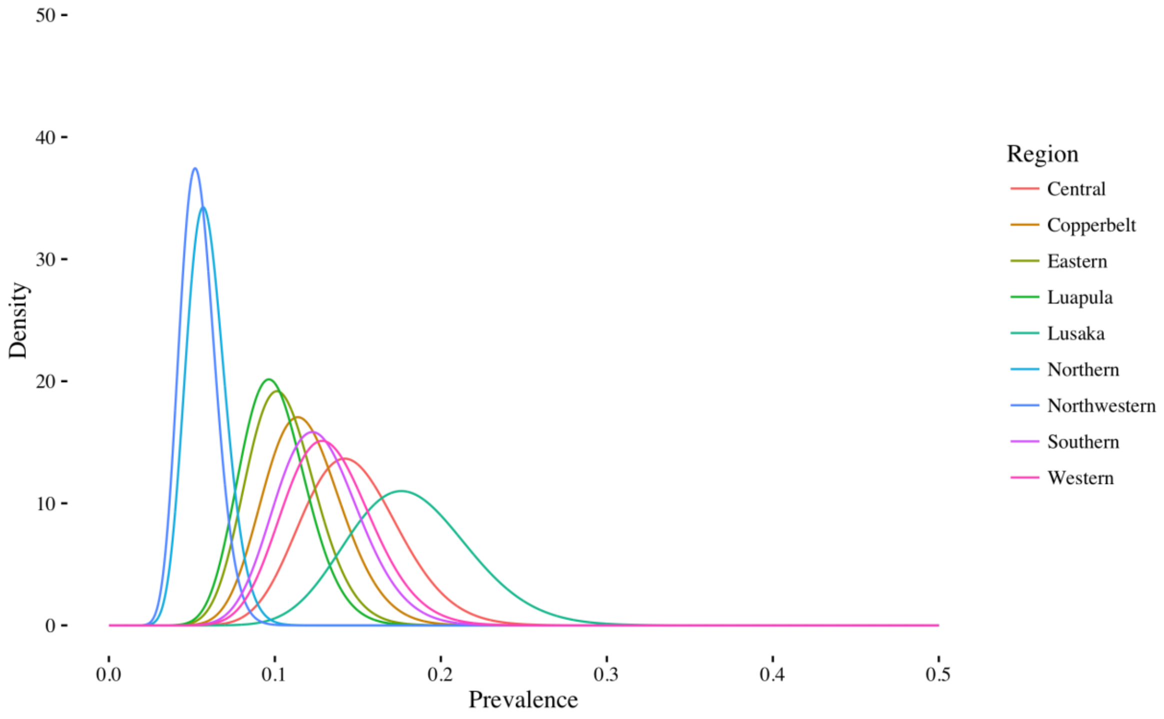

Figure S2(b): Uncertainty in HIV prevalence estimates by region

Figure 3 shows the trends in the epidemic predicted by the model over the study period. These outputs represent an investment strategy in which the baseline scenario is continued (i.e. 80% of new late stage infections receive ART and there is no active circumcision intervention or provision of early ART). The rapid improvements in outcomes observed in the year 2006 (or spike in outcomes) reflect the initial scale-up to 80% of ART among prevalent cases of HIV with CD4 counts below 350 in this year. We included the assumption that provision of late ART is scaled up until all those with late stage disease receive it (reflecting typical late ART scale up to universal coverage levels of 80%). In the year 2006, there is an immediate scale up of ART treatment for those with late stage infections. This means that the proportion of prevalent late stage infections receiving ART in the year 2006 suddenly becomes 80%. Of course, the model could provide for a gradual scale-up, but this makes no difference to the outcomes of interest here. From 2006-2015, there is a gradual scale up in the proportion of incident late stage infections who receive ART from 0% - 80%. From 2015 onwards, the model focuses on the policy intervention choices (early ART, circumcision, late ART) and the impact of these choices over the period 2015-2030. Therefore, the main findings of the model are driven by what happens after 2015 since this is when the interventions are introduced.

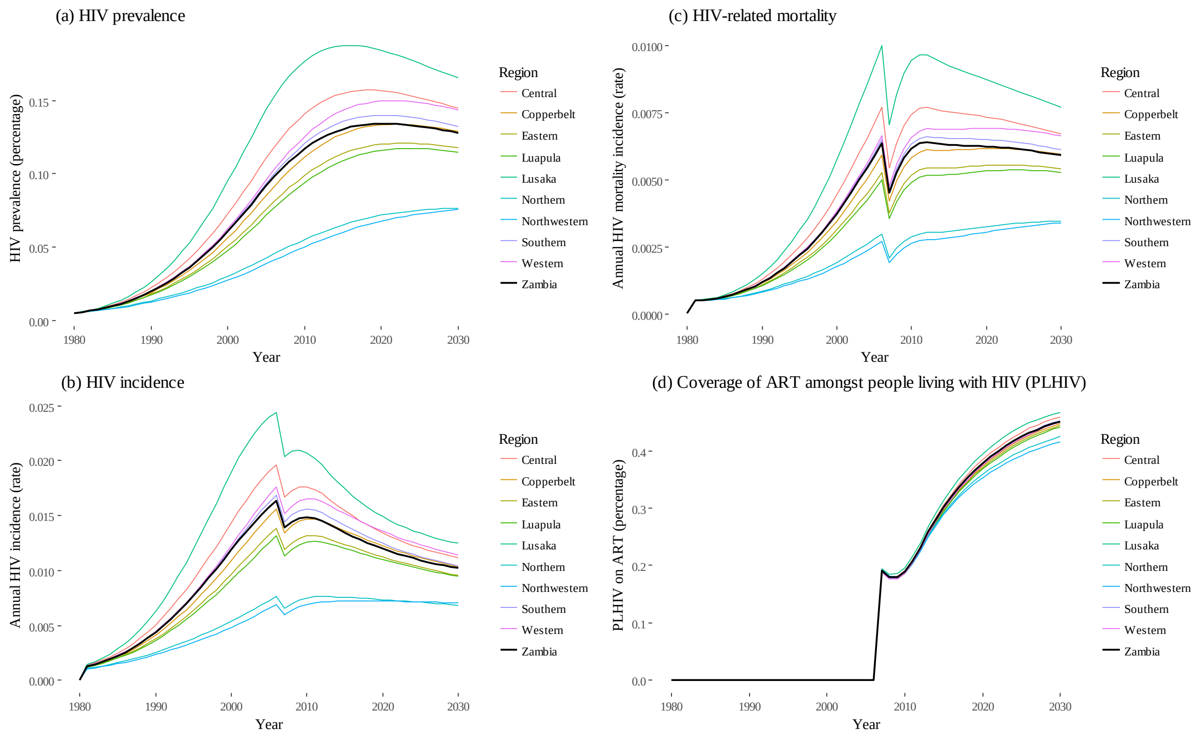

Figure S3: Model projections for modelled population (adults aged 15-49)

2 Detailed description of resource allocation model

We represent resource allocation as a two-stage decision making process.(17) The first stage involves planning coverage levels for each intervention in each region, and the corresponding resources that will be allocated. Decision makers are assumed to base their plans on the expected costs and health benefits of all possible ways in which the resources could be allocated. We assume that decision makers will select the resource allocation that maximises health across regions subject to the national HIV budget.

The expected costs and health benefits used in this process reflect the average costs and health benefits across all possible eventualities that could occur (or across all 'realisations' of uncertainty). In reality, only one of these eventualities occurs and the decision maker must react to this. If they don't and instead stick to their original plans then they may end up exceeding allocated budgets or being left with unspent funds. We therefore model a second stage in which decision makers respond to the realised eventuality. These two stages are described in detail in the following sections.

We run resource allocation model for a range of budgets. Resch et al (18) have estimated total AIDS expenditure for 12 low- and middle- income countries (Botswana, Côte d'Ivoire, Ethiopia, Kenya, Mozambique, Namibia, Nigeria, Rwanda, South Africa, Tanzania, Uganda, and Zambia). These countries account for 52% of AIDS cases worldwide. Using the most recent data available for each country (which ranged from 2009/10 to 2012/13) in combination with population data from the World Bank (4) implies a total annual HIV budget of $3-$175 per capita. Ten of the twelve countries had a total annual HIV budget of $3-$32 per capita. The remaining countries - Botswana and Namibia - had much higher annual budgets of $175 and $136 per capita respectively. We therefore explored annual budgets of $3-32 per capita, scaling these up to reflect the total population in our example at the start of the intervention period and the 15 year time horizon. This resulted in a budget range of $0-$3,000 million. We present results in this paper for budgets of $0-$2,200 million as at budgets of $2,200 million all modelled investment opportunities had been exhausted.

2.1 Planning the HIV investment strategy

We constructed an optimisation model to identify the allocation of resources across geographical areas and interventions that maximises population health. This model selects coverage levels for each intervention (ϕ_L, ϕ_E and ϕ_C) in order to maximise total health benefits (QALYs) subject to expected total costs being within the HIV budget. As the impact of the interventions is interdependent we model each possible combination of coverage

levels for early ART, late ART and circumcision. A region could implement any combination of coverage levels, we refer to each combination as an '*intervention set*'.

The optimisation model takes the form of a linear integer mathematical programme, the goal is to maximise the objective function:

$$\max_X \sum_{j=1}^{J} \sum_{i=1}^{I} H_{ij}\, x_{ij}$$

Subject to three constraints:

$x_{ij} \in (0,1)$ [constraint 1]

$\sum_{i=1}^{I} x_{ij} = 1 \text{ for j} = 1, \ldots, \text{J}$ [constraint 2]

$\sum_{j=1}^{J} \sum_{i=1}^{I} C_{ij}\, x_{ij} \leq B$ [constraint 3]

where J denotes the number of regions $(j = 1, \ldots, J)$ and I denotes the number of possible intervention sets $(i = 1, \ldots, I)$. The set of decision variables is defined by $X = (x_{ij}, i = 1, \ldots, I;\ j = 1, \ldots, J)$, where x_{ij} represents the binary choice of whether or not intervention set i is selected for region j. The expected health outcomes, and costs associated with each intervention set in each region are denoted by H_{ij} and C_{ij}, respectively.

The objective function specifies that the goal is to maximise total health across regions. The optimisation process selects values for the decision variables (x_{ij}) that will maximise total health subject to three constraints: (1) each decision variable must take the value 0 or 1 i.e. an intervention set can be selected or not; (2) within each region only one intervention set can be selected; and (3) the total cost of the selected intervention sets across regions must not exceed the national budget (B).

In addition, the decision maker faces a constraint that universal access to late ART must be offered to all those who can feasibly be reached for treatment (80% of all late stage infections (1)), and where universal access is unaffordable, availability of late ART should be the same across regions. The coverage levels for late ART, circumcision, and early ART associated with each intervention set are $\phi_E[x_{ij}]$, $\phi_L[x_{ij}]$ and $\phi_C[x_{ij}]$ respectively. The first component of the additional constraint is:

$$\frac{1}{J}\sum_{j=1}^{J}\sum_{i=1}^{I} x_{ij} \cdot I\big(\phi_L[x_{ij}] = 0.80\big) - \sum_{i=1}^{I} x_{ij} \cdot I\big(\phi_E[x_{ij}] + \phi_C[x_{ij}] > 0\big) \geq 0 \;\; for\, j = 1, \ldots, J$$

where I denotes an indicator function that takes a value of 1 if the condition is true and zero otherwise. This constraint requires that the all regions must have implemented late ART at 80% (first term) if a region has implemented early ART or circumcision (second term).

The second component requires that coverage of late ART is equal across regions:

$$\frac{1}{J}\sum_{j=1}^{J}\sum_{i=1}^{I} \phi_L[x_{ij}] - \sum_{i=1}^{I} \phi_L[x_{ij}] = 0\, for\, j = 1, \ldots, J$$

This is achieved by requiring the average late ART coverage (first term) to equal the regional late ART coverage (second term) for every region.

2.2 Responding to the realised state of the world

We model a series of different policy responses to each realisation of uncertainty. As we do not know which realisation will occur, the outcomes associated with each policy are calculated for each realisation and then averaged across all realisations.

2.2.1 Regional policy under current information

Under the regional policy under current information each region is required to remain within their planned regional budget. To achieve this spending on each intervention in each region is maintained at the planned level. This does not guarantee that regions will achieve the coverage they planned. This is illustrated in Figure S4 which shows the response to uncertainty for a single intervention in a single region. Based on expected costs and effects the decision maker plans to implement 60% coverage at a cost of $1 million. Panel (a) of Figure S4 shows that if actual costs are higher than expected (e.g. due to a larger number of individuals presenting for treatment, or higher than expected treatment costs) the decision maker must cut coverage to 30% to remain within budget. When actual costs are lower than expected, coverage is expanded (Figure S4 panel (b)). If the available budget exceeds the amount required to fund the maximum possible coverage (here 80%), then the difference between the available funds and the cost of achieving maximum coverage is wasted.

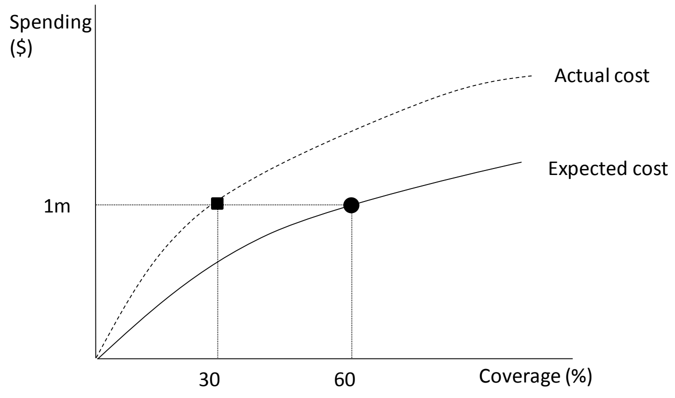

(a) Response to actual costs exceeding expected costs

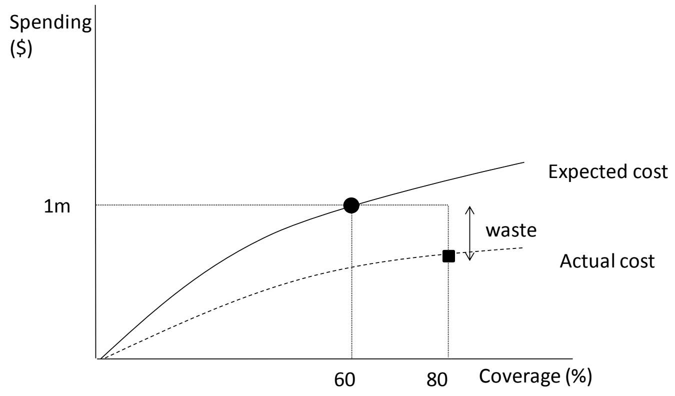

(b) Response to actual costs falling below expected costs

Figure S4: Regional budget policy (one region, one intervention): Circles indicate planned coverage, squares indicate realised coverage. Panel (a) shows that when costs exceed those expected coverage must be reduced. Panel (b) shows that when costs are lower than expected coverage can be expanded. If the original planned spending exceeds the cost of maximum coverage (here 80%) funds will be wasted.

2.2.2 Perfect information

It is possible to improve the information available to inform resource allocation decisions. For example, large surveys could be conducted to improve our understanding of regional epidemiological conditions. We assess the maximum possible value of improving the information available to decision makers by looking at the health we could generate if we had ‘perfect’ information. If we had perfect information we would know exactly which eventuality would occur and could perform the initial optimisation described in Section 2.1 using observed costs and benefits (rather than expected costs and benefits). To establish the value of perfect information we therefore run the optimisation analysis under each realisation of uncertainty and average the results across all realisations of uncertainty.

2.2.3 National policy under current information

Under the national policy plans are only revised to the extent that national HIV costs under- or over-run the national HIV budget. Under this policy funds are fungible across interventions and regions to support planned coverage levels. Any surplus at the national level is used to increase funding beyond that required to meet the

original plans by the same proportion for each intervention in each region. Any deficit at the national level results in the funds required to achieve planned coverage being cut by the same proportion.

The implications of the national policy are shown in Figure S5 where we have two interventions and one is highly cost-effective (A) whereas the other is less cost-effective (B). Under the regional policy the decision maker plans to implement 60% coverage for both interventions but actually delivers coverage of 45% for A and 70% for B (Figure S5 Panel (a)). Under a national policy (Figure S5 Panel (b)) plans are identical to those made under the regional policy. Although the cost of interventions A and B are not as expected, the total costs are within the originally planned total cost. Funds can therefore be re-allocated between regions to achieve the original planned coverage levels of 60% for intervention A and B.

2.2.4 Contingency fund policy under current information

When a contingency fund is used, plans are made based on the total HIV budget less the amount dedicated to the contingency fund. Plans are therefore more modest than under other policies. The contingency fund exists to avoid programmes being cut back due to funding issues. If there are sufficient funds to pay for the planned programme then this policy works in exactly the same way as the regional policy though with a lower budget. When there are insufficient funds to pay for the planned programme the contingency fund can be used. If the contingency fund is insufficient to pay for all programme over-runs, a reduced claim is met (and claims are reduced by the same proportion across all programmes).

If implemented for one intervention in one region the contingency fund will always result in lower coverage and health than the regional policy. This is because when a contingency fund operates spending on the intervention will always be equal to, or lower than, spending under the regional policy.[1] However, when there are multiple interventions or regions the contingency fund can result in improved health compared to the regional policy. Under the contingency fund 20% of the total $3m budget ($0.6m) is set aside as contingency (Figure S5 Panel (c)). This results in a lower investment in intervention B (as this is less cost-effective than intervention A) which has a planned coverage of 40%, and an actual coverage of 45%. The cost over-run for intervention A is met by the contingency fund, allowing planned coverage to be preserved at 60%. In this instance the contingency fund may improve population health compared to the regional policy as it supports coverage of the more cost-effective intervention. This will not be the case in all realisations of uncertainty. The contingency fund may produce less health than the fixed regional budget policy under a range of conditions. If there are under-runs in a number of programmes this will mean that the contingency fund is partially or completely unspent. If the contingency fund preserves planned programmes in states of the world in which they are particularly cost-ineffective (e.g. due to high costs) then this would also reduce health. Finally, if the contingency fund results in plans that involve maximum coverage for more interventions this will increase the likelihood of wasted funds.

[1] This, can be shown by looking at the expenditure on the intervention in each scenario:
Regional policy = min(total budget, spend required to deliver maximum coverage),
Contingency fund, under-run = min(total budget – contingency fund, spend required to deliver maximum coverage),
Contingency fund, over-run = min(total budget, spend required to deliver planned coverage).

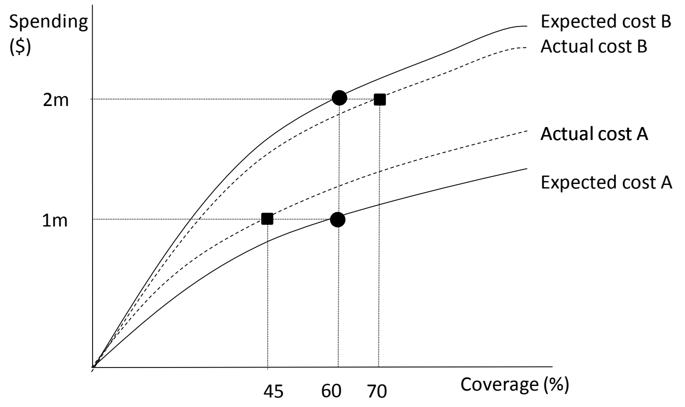

(a) Regional policy

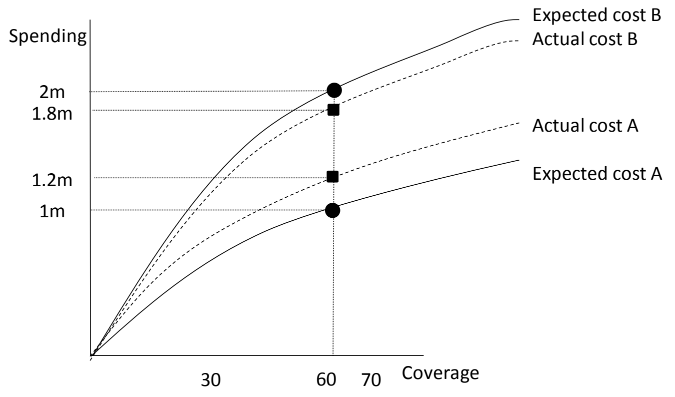

(b) National policy

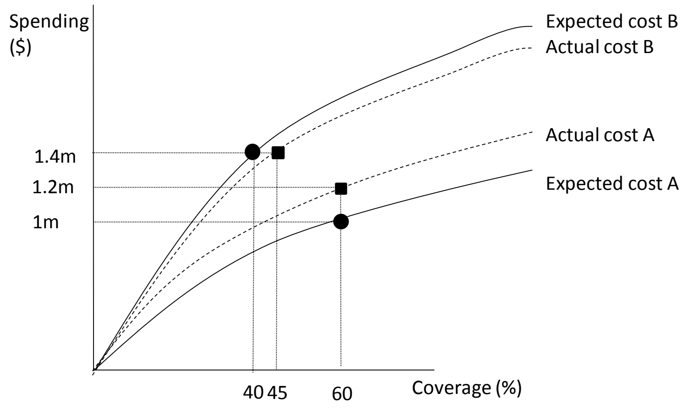

(c) Contingency fund policy

Figure S5: Comparison of regional policy, national policy and contingency fund policy (one region, two interventions): Circles indicate planned coverage, squares indicate realised coverage.

3 Implementation of simulation modelling

Estimating the health generated under perfect information is relatively straightforward. To estimate the health expected under perfect information the optimisation process is run for each realisation of uncertainty and the corresponding health is recorded. The expected health is then estimated as simply the average across all realisations of uncertainty. The implementation is more complex for the current information scenarios. In each of these scenarios the response to uncertainty is either to preserve or adjust spending. There is therefore a pattern of spending on each intervention in each region that we expect for each realisation of uncertainty. However, spending on each intervention is not an input in the transmission model. A matching process is therefore used to identify the intervention set that most closely produces the spending pattern we expect (in each region). This allows the health associated with the spending pattern to be estimated. Again, this is estimated for each realisation of uncertainty, and then averaged across all realisations of uncertainty.

The transmission model is run for all intervention sets defined by evaluating interventions at 1% increments (ART interventions) and 2% increments (circumcision intervention) in coverage levels ($\phi_L = 0\%, 1\%, \dots, 80\%$; $\phi_C = 0\%, 2\%, \dots, 40\%$; and $\phi_E = 0\%, 1\%, \dots, 73\%$). In total 124,173 intervention sets (81*21*73) are therefore evaluated in each region and for each realisation of uncertainty. The exact spending pattern of interest will generally not have been observed in this set of transmission model runs. The intervention set that most closely matches the required spending pattern is therefore identified using a two stage process. Firstly, the set of 'affordable' interventions are identified. Affordable interventions are those which offer spending on each intervention component (late ART, circumcision and early ART) that is less than or equal to that of interest. Secondly, amongst these affordable intervention sets the absolute difference between spending on each intervention and the spending expected is calculated. These absolute differences are then summed across the three interventions and the intervention set with the smallest sum of absolute differences is selected as the best match. In some regions of Zambia the proportion of individuals entering the modelled population who are already circumcised is relatively high (see Table S1). Under all policies the maximum intervention level for circumcision is set so that the total population coverage for circumcision cannot exceed 40% for all individuals (i.e. 80% for men), this results in variation across regions in the extent to which circumcision coverage can be increased by the HIV programme.

4 Additional results: size of the contingency fund

The contingency fund could theoretically be set at any size between 0 and 100% of the total budget. We explored contingency funds of 5 to 50% of the budget in 5% increments. The health generated by different contingency funds is shown in Figure S6 for four national budgets. The optimal size of the contingency fund will depend on the context and the national HIV budget. As the size of the contingency fund expands the benefits it offers in terms of insurance increase, however it becomes less likely that all contingency funds will be needed and funds are therefore more likely to be wasted. Furthermore, as the contingency fund expands the opportunity cost of the fund also increases as more and more valuable investment plans are displaced. In our analysis, where contingency funds did improve health (i.e. at higher national HIV budgets), funds of around 5% of the total HIV budget generated the most health and are therefore presented in the main text.

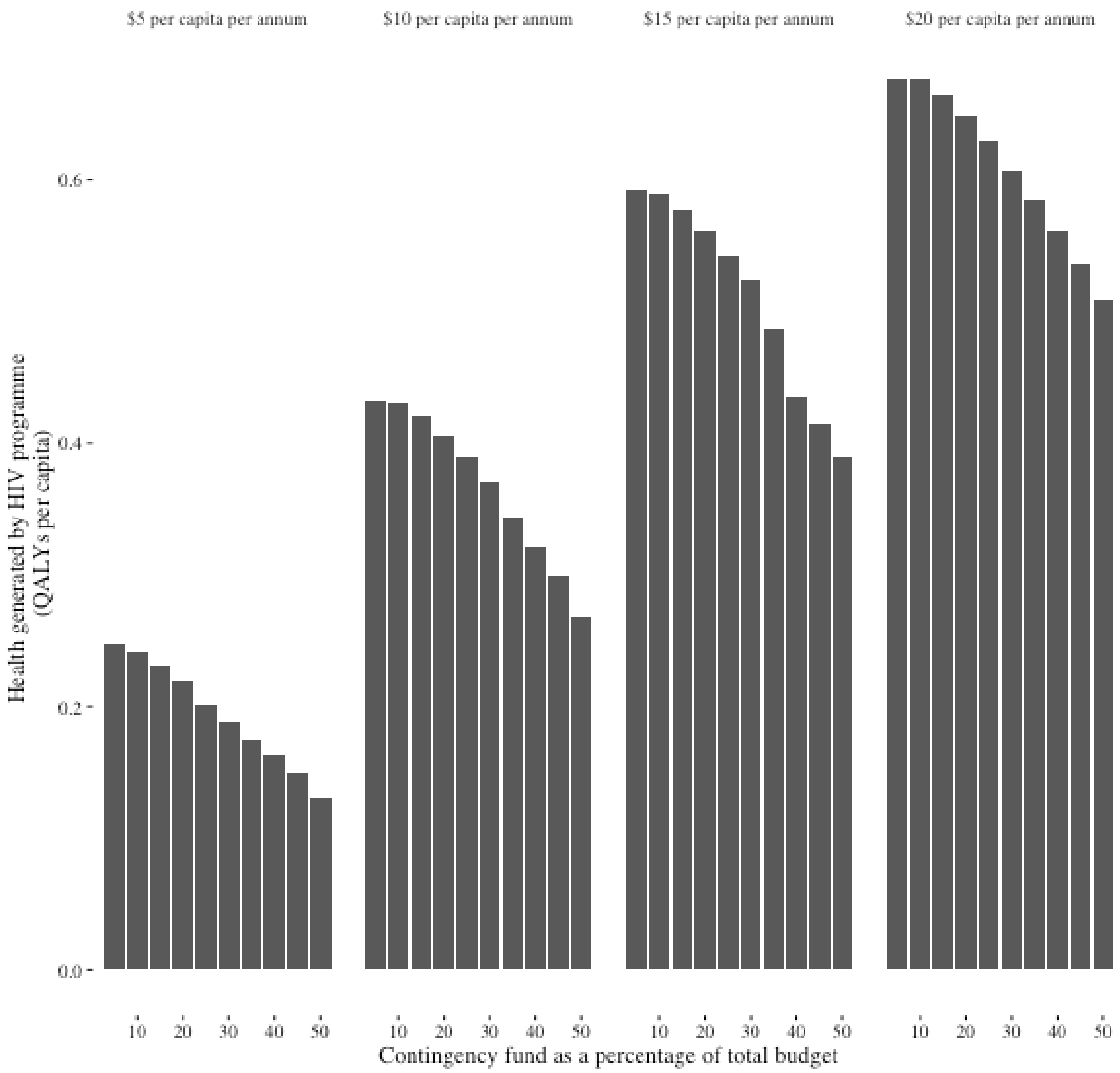

Figure S6: Health generated by contingency funds of 5-50% of total national HIV budget, at a range of national HIV budgets

5 Software and code

All modelling was conducted in the statistical programming software R, version 3.2.2 (19) , the package data.table (20) was used to manage and manipulate large datasets. Optimisation of the linear integer program was performed using Gurobi software version 6.0.4 called from R (21). The code below shows provides key excerpts from the resource allocation code to facilitate use by other researchers.

```
###########################################################################
#Part 1: Create function to identify health-maximising HIV investment
strategy

LP<-function(h,b,descriptors){

#The arguments of the function are h= vector of health benefits for each
combination of coverage levels for each region; b=vector of costs for each
combination of coverage levels for each region; descriptors= matrix of the
corresponding coverage levels for each intervention (labelled IntA, IntB,
IntC) and intervention level costs (meancostA, meancostB, meancostC).
```

```
  #generate a model object
        model<-list()
  #assign this as a maximisation problem
        model$modelsense<-"max"
  #assign the choice variable as binary i.e. each combination of coverage levels can either be implemented or not within a region.
        model$vtype<-'B'
  #set objective function as health
        model$obj<-c(t(h))
  #set matrix of constraints
  #specify the dimensions of the matrix
      model$A<-
      matrix(rep(NA,no.interventions*(no.regions+1)*no.regions),nrow=1+no.regions)
  #set the left hand side of the constraints (i.e. the coefficients on the binary choice variables)
  #constraint 1: budget cannot be exceeded
  model$A[1,]<-c(t(b))
  #constraint 2: each region can only implement one set of coverage levels
      model$A[2:(no.regions+1),]<-
      matrix(c(rep(c(rep(1,no.interventions),rep(0,no.interventions*no.regions)),no.regions-1),
      rep(1,no.interventions)),byrow=TRUE,ncol=as.numeric(no.interventions*no.regions))
  #specify sign of the constraints
      model$sense <- c('<=', rep(c('='),no.regions))
    #specify right hand side of the constraints (where budget represents the total national HIV budget available across regions)
      model$rhs <- c(budget, rep(c(1),no.regions))

   #add entitlement constraint
   #set the left hand side of the constraints
      model$A<-rbind(model$A,model$A[2:(no.regions+1),]*matrix(rep((-no.regions)*((descriptors$IntA+descriptors$IntC)>0),no.regions),nrow=no.regions,byrow=TRUE)+
      matrix(rep(descriptors$IntB==0.8,no.regions),nrow=no.regions,byrow=TRUE),model$A[2:(no.regions+1),]*matrix(rep(descriptors$IntB,no.regions),nrow=no.regions,byrow=TRUE)-
      matrix(rep(descriptors$IntB,no.regions)/no.regions,nrow=no.regions,byrow=TRUE))

   #specify sign and right hand side of the constraints
      model$sense<-c(model$sense,rep(">=",no.regions),rep("=",no.regions))
      model$rhs<-c(model$rhs,rep(0,no.regions),rep(0,no.regions))

  #constraint: decision options that would result in circumcision coverage of more than 40% cannot be selected

  region_circ<-
c(0.02,0.08,0.01,0.05,0.05,0.02,0.36,0.01,0.19)[order(c(0.147, 0.118, 0.105,0.100, 0.182, 0.059, 0.054, 0.127, 0.133))]
  not_allowed<-((circ_level+rep(region_circ,each=no.interventions))>0.4)
  model$A<-
rbind(model$A,model$A[2:(no.regions+1),]*matrix(rep(not_allowed,no.regions),nrow=no.regions,byrow=TRUE))
  model$sense<-c(model$sense,rep("=",no.regions))
  model$rhs<-c(model$rhs,rep(0,no.regions))

   #run optimisation model
      results<-gurobi(model,params)
```

```
  #extract results (x represents the binary choice regarding the
combination of coverage levels to use in each region)
  x<-results$x

  #store results
  #note bregion is expected total expenditure at the regional level, B is
the equivalent at the national level
  choice<-
data.table(Region=1:9,descriptors[x==1,],bregion=b[x==1],qalys=h[x==1],B=su
m(b[x==1]))

setnames(choice,names(choice),c(names(choice)[1],c("planA","planB","planC",
"meancostA","meancostB","meancostC"),names(choice)[8:10]))
  return(choice)
}
```

##

#Part 2: Run this for each budget using expected costs and QALYs in order to determine the planned investment strategy

```
#read in file containing expected (across all simulations) costs and
outcomes associated with each possible coverage combination in each region
as output from analysis of transmission model.
expected<-fread("expected.csv",header=F,sep=",")
setnames(expected,old=c("Region","RegionPrev","IntA","IntB","IntC","meancos
tA","meancostB","meancostC","meancost","meanQALYs"))
circ_level<-expected[,.(IntC)]

#set national HIV budgets
budgets<-(seq(0,2.5E+09,2.27E+07))
#extend budgets to include those that form the basis of investment planning
under the contingency policy
  surplus<-seq(0,50,5)/100
  budgets<-(1-surplus)*budgets
  no.budgets<-length(budgets)

#create matrix to record choices made in HIV investment plan
myopic_allbudgets<-
data.frame(matrix(rep(NA,no.regions*no.budgets*11),ncol=11))
colnames(myopic_allbudgets)<-
c("Region","planA","planB","planC","meancostA","meancostB","meancostC","bre
gion","meanQALYs","B","National_Budget")

for (j in 1:no.budgets) {
  budget<-budgets[j]
  #run optimisation (LP function from Part 1) to identify optimal choice
  myopic_choice<-
LP(h=expected$meanQALYs,b=expected$meancost,descriptors=expected[,.(IntA,In
tB,IntC,meancostA,meancostB,meancostC)])
  setkey(myopic_choice,Region)
  myopic_choice<-cbind(myopic_choice,National_Budget=budget)

    if(j==1) {myopic_allbudgets[1:9,]<-myopic_choice} else {myopic_
allbudgets<-cbind(myopic_allbudgets,myopic_choice)}
    rm(myopic_choice)
}
```

##

#Part 3: Run model for each realisation of uncertainty and identify health generated under different policy scenarios

```
#For each realisation of uncertainty (where sims represents an index number for each realisation)
for(i in 1:length(sims)) {
iter<-sims[i]

#load in transmission data for that simulation, where transmission data contains the iteration index,
#the values of uncertain parameters (prev13, epsilonart, epsiloncirc, artcost and circcost) for each region, as well as all possible combinations of coverage levels and the associated costs and qalys
#where costs and qalys represent incremental costs and qalys compared to no coverage of any intervention
data_i<-
fread(paste("Transmission_sim",iter,".csv",sep=""),header=F,sep=",")
setnames(data_i,old=c("Iter","IntA","IntB","IntC","Region","prev13","epsilonart","epsiloncirc","artcost","circcost","costA","costB","costC","totalcost","qalys"))
setkey(data_i,Region,IntA,IntB,IntC)

#identify budgets, regions and policies
budgets<-myopic_allbudgets[,unique(National_Budget)]
no.budgets<-length(budgets)
policies<-c("Soft budget","National coverage","Regional spend","Perfect information")
no.policies<-length(policies)

#budgets of interest for non-contingency analyses, note these must be a subset of those examined in the myopic analysis
no_surplus_budgets<-(seq(0,2.5E+09,2.27E+07))
no.no_surplus_budgets<-length(no_surplus_budgets)

#Set budgets for contingency policy
##percentage surplus investigated
surplus<-seq(5,50,5)/100
no.surplus<-length(surplus)
surplus_policies<-data.frame(budget=rep(no_surplus_budgets[-1],each=no.surplus))
surplus_policies$surplus<-surplus_policies$budget*surplus
surplus_policies$decision_budget<-surplus_policies$budget-surplus_policies$surplus

#file to store iteration-specific information
results_i<-
data.frame(matrix(rep(NA,no.regions*no.policies*no.no_surplus_budgets*12),ncol=12))
results_i[,1]<-rep(no_surplus_budgets,each=no.regions*no.policies)
results_i[,2]<-rep(policies,each=no.regions)

##for surplus policy
# *2 below as regional coverage and national coverage versions of surplus policies
results_surplus_i<-
data.frame(matrix(rep(NA,no.regions*no.surplus*(no.no_surplus_budgets-1)*12),ncol=12))
results_surplus_i[,1]<-rep(surplus_policies$budget,each=no.regions)
```

```
results_surplus_i[,2]<-paste(rep(surplus*100,each=no.regions),"%
surplus",sep="")

#Function to determine health generated under different policies
##Note SpendPlan denotes cost of original plans, bregion=planned regional
budget, B is planned national budget, and Cost_PlanReg = cost of original
plans for A+B+C
scenarios1to4<-function(selected_policy) {
  #Depending upon scenario selected calculate target spend
    if(selected_policy=="National coverage") {
      data[, ':=' (SpendTargetA = Cost_PlanA*B/Cost_PlanNat,
      SpendTargetB = Cost_PlanB*B/Cost_PlanNat,
      SpendTargetC = Cost_PlanC*B/Cost_PlanNat),by=.(Region)]
    } else if(selected_policy=="surplus") {
      data[, ':='
(SpendTargetA=(as.numeric(meancostA)>=Cost_PlanA)*as.numeric(meancostA)+

(as.numeric(meancostA)<Cost_PlanA)*(as.numeric(meancostA)+(Cost_PlanA-
as.numeric(meancostA))*min(1,surplus_current/total_claims)),

SpendTargetB=(as.numeric(meancostB)>=Cost_PlanB)*as.numeric(meancostB)+

(as.numeric(meancostB)<Cost_PlanB)*(as.numeric(meancostB)+(Cost_PlanB-
as.numeric(meancostB))*min(1,surplus_current/total_claims)),

SpendTargetC=(as.numeric(meancostC)>=Cost_PlanC)*as.numeric(meancostC)+

(as.numeric(meancostC)<Cost_PlanC)*(as.numeric(meancostC)+(Cost_PlanC-
as.numeric(meancostC))*min(1,surplus_current/total_claims))),by=.(Region)]
    } else {
      data[, ':=' (SpendTargetA = as.numeric(meancostA),
      SpendTargetB = as.numeric(meancostB),
      SpendTargetC = as.numeric(meancostC)),by=.(Region)]
    }

  #set NaN values generated by dividing by zero by zero (when plans are
zero, Cost_PlanReg and Cost_PlanNat are zero)
  data[is.nan(SpendTargetA), SpendTargetA := 0]
  data[is.nan(SpendTargetB), SpendTargetB := 0]
  data[is.nan(SpendTargetC), SpendTargetC := 0]

  #Calculate discrepancy
  data[,':='(DiscrepA = costA-SpendTargetA,DiscrepB = costB-
SpendTargetB,DiscrepC = costC-SpendTargetC),by=.(Region)]
  #compute sum of absolute discrepancies
  data[, SE := abs(DiscrepA)+abs(DiscrepB)+abs(DiscrepC)]
  #find minimum absolute discrepancy for each region and each realisation
of uncertainty
  #note that this has to be within the spendtargets
  data[,minSE := min(SE[costA<=SpendTargetA & costB<=SpendTargetB &
costC<=SpendTargetC]),by=.(Region)]
  #store information associated with closest spend
  scenario_outcomes<-
data[SE==minSE,.(Region,IntA,IntB,IntC,costA,costB,costC,totalcost,qalys,sp
ent_surplus=(selected_policy=="surplus")*(B+min(surplus_current,total_claim
s)))]
  return(scenario_outcomes)
}

for (j in 1:no.budgets) {
  budget<-budgets[j]
```

```
  #Merge iteration data with myopic_choice for that budget
  data<-merge(data_i,myopic_choice[National_Budget==budget,],by="Region")
  #Calculate cost associated with original plan under realisation of
uncertainty in each region
  data[, ':=' (Cost_PlanA = costA[IntA==planA & IntB==planB & IntC==planC],
               Cost_PlanB = costB[IntA==planA & IntB==planB & IntC==planC],
               Cost_PlanC = costC[IntA==planA & IntB==planB &
IntC==planC]),by=.(Region)]
  #Total regional and national spend associated with planned coverage
  data[,Cost_PlanReg :=Cost_PlanA+Cost_PlanB+Cost_PlanC, ]
  data[,Cost_PlanNat := sum(Cost_PlanReg),by=.(IntA,IntB,IntC)]

  #Extra calculations for contingency policy
  #calculate total claims on surplus
  data[,total_claims := max(Cost_PlanA-meancostA,0)+max(Cost_PlanB-
meancostB,0)+max(Cost_PlanC-meancostC,0),by=.(Region)]
  data[,total_claims := sum(total_claims),by=.(IntA,IntB,IntC)]

# remove decision options which would result in circumcision coverage of
more than 40%
      data_all<-data
      circ_level<-as.matrix(data[,.(IntC)])
      region_circ<-
c(0.02,0.08,0.01,0.05,0.05,0.02,0.36,0.01,0.19)[order(c(0.147, 0.118,
0.105,0.100, 0.182, 0.059, 0.054, 0.127, 0.133))]
      allowed<-(1-
((circ_level+rep(region_circ,each=no.interventions))>0.4))
      data<-data[allowed[,1]==1]

  #Assign policy outcomes for each policy with the exception of the
contingency policies
  if(budget%in%no_surplus_budgets) {
    for (k in 1:no.policies) {
        policy_outcome<-
data.frame(matrix(rep(NA,no.regions*10),nrow=no.regions))
        if(policies[k]=="Soft budget") {
          policy_outcome[,c(1:9)]<-data[IntA==planA & IntB==planB &
IntC==planC,

.(Region,IntA,IntB,IntC,costA,costB,costC,totalcost,qalys)] } else
if(policies[k]=="National coverage") {
policy_outcome<-scenarios1to4("National coverage")} else
if(policies[k]=="Regional spend")  {
policy_outcome<-scenarios1to4("Regional spend")} else {

#for perfect information the optimisation is re-run for the current
iteration
policy_outcome[,c(1:9)]<-LP(data_all[,qalys],data_all[,totalcost],data_all[

,.(IntA,IntB,IntC,costA,costB,costC,treatedA,treatedB,treatedC)],constraint
_current,ent=entitlement_current)[,c(1:9),with=F]
                                                  }
        results_i[results_i$X1==budget &
results_i$X2==policies[k],c(3:12)]<-policy_outcome
      }
  }

  #Assign policy outcomes for the contingency policy
  #establish the number of surplus policies associated with a the current
decision budget
```

```
  no.surplus_current<-
dim(surplus_policies[surplus_policies$decision_budget==budget,])[1]
  if (no.surplus_current!=0) {
    for (l in 1:no.surplus_current) {
      surplus_current<-
surplus_policies$surplus[surplus_policies$decision_budget==budget][l]
      surplus_name<-paste(surplus_current/(surplus_current+budget)*100,"%
surplus",sep="")
      results_surplus_i[results_surplus_i$X1==(budget+surplus_current) &
results_surplus_i$X2==surplus_name,c(3:12)]<-scenarios1to4("surplus")
    }
  }
}

#combine results for all policies and then for all iterations
results_withsurplus<-cbind(rbind(results_i,results_surplus_i),iter)
if(i==1) {results_withsurplus_all<-results_withsurplus} else
{results_withsurplus_all<-
rbind(results_withsurplus_all,results_withsurplus)}
}


#######################################################################################
```

6 Supplementary material references

References

1. World Health Organization. Global update on HIV treatment 2013: results, impact and opportunities. 2013.
2. HIV/AIDS JUNPo, HIV/Aids JUNPo. 90-90-90: an ambitious treatment target to help end the AIDS epidemic. Geneva: UNAIDS. 2014.
3. Meyer-Rath G, Over M, Klein DJ, Bershteyn A. The Cost and Cost-Effectiveness of Alternative Strategies to Expand Treatment to HIV-Positive South Africans: Scale Economies and Outreach Costs. Center for Global Development Working Paper. 2015(401).
4. Population growth (annual %) [Internet]. 2015 [cited 08/02/2015]. Available from: http://data.worldbank.org/indicator/SP.POP.GROW.
5. McGillen JB, Anderson S-J, Dybul MR, Hallett TB. Optimum resource allocation to reduce HIV incidence across sub-Saharan Africa: a mathematical modelling study. The Lancet HIV. 2016;3(9):e441-e8.
6. Lodi S, Phillips A, Touloumi G, Geskus R, Meyer L, Thiébaut R, et al. Time from human immunodeficiency virus seroconversion to reaching CD4+ cell count thresholds< 200,< 350, and< 500 Cells/mm3: assessment of need following changes in treatment guidelines. Clinical infectious diseases. 2011;53(8):817-25.
7. Siegfried N, Muller M, Deeks JJ, Volmink J. Male circumcision for prevention of heterosexual acquisition of HIV in men. The Cochrane Library. 2009.
8. Baggaley RF, White RG, Hollingsworth TD, Boily M-C. Heterosexual HIV-1 infectiousness and antiretroviral use: systematic review of prospective studies of discordant couples. Epidemiology (Cambridge, Mass). 2013;24(1):110.
9. Anderson S-J, Cherutich P, Kilonzo N, Cremin I, Fecht D, Kimanga D, et al. Maximising the effect of combination HIV prevention through prioritisation of the people and places in greatest need: a modelling study. The Lancet. 2014;384(9939):249-56.
10. Bautista-Arredondo S, Sosa-Rubí SG, Opuni M, Contreras-Loya D, Kwan A, Chaumont C, et al. Costs along the service cascades for HIV testing and counselling and prevention of mother-to-child transmission. AIDS (London, England). 2016;30(16):2495.
11. Martin G, Bollinger L, Pandit-Rajani T, Forsythe S, Stover J. Costing male circumcision in Zambia and implications for the cost-effectiveness of circumcision as an HIV intervention. 2007.
12. Sosa-Rubi S, Bautista-Arredondo S, Opuni M, Contreras-Loya D, La Hera-Fuentes G, Salas-Ortiz A, et al., editors. Average costs of voluntary medical male circumcision and their determinants in Kenya, Rwanda, South Africa and Zambia. JOURNAL OF THE INTERNATIONAL AIDS SOCIETY; 2016: INT AIDS SOCIETY AVENUE DE FRANCE 23, GENEVA, 1202, SWITZERLAND.
13. Tagar E, Sundaram M, Condliffe K, Matatiyo B, Chimbwandira F, Chilima B, et al. Multi-country analysis of treatment costs for HIV/AIDS (MATCH): facility-level ART unit cost analysis in Ethiopia, Malawi, Rwanda, South Africa and Zambia. PloS one. 2014;9(11):e108304.
14. Guthrie T, Moyo C, Kinghorn A, Kuehnle J, Sinyangwe G, van Rensberg C, et al. Costs and Outcomes of ART scale-up in Zambia: Modelled Estimates for Test and Treat & Community Based Service Delivery Models. Policy Brief, September 2016. . Johannesburg: USAID EQUIP Policy Brief, 2016.: 2016.
15. Sgaier SK, Baer J, Rutz DC, Njeuhmeli E, Seifert-Ahanda K, Basinga P, et al. Toward a systematic approach to generating demand for voluntary medical male circumcision: Insights and results from field studies. Global Health: Science and Practice. 2015;3(2):209-29.
16. Bollinger L, Adesina A, Forsythe S, Godbole R, Reuben E, Njeuhmeli E. Cost drivers for voluntary medical male circumcision using primary source data from sub-Saharan Africa. 2014.
17. McKenna C, Chalabi Z, Epstein D, Claxton K. Budgetary policies and available actions: a generalisation of decision rules for allocation and research decisions. Journal of health economics. 2010;29(1):170-81.

18. Resch S, Ryckman T, Hecht R. Funding AIDS programmes in the era of shared responsibility: an analysis of domestic spending in 12 low-income and middle-income countries. The Lancet Global Health. 2015;3(1):e52-e61.
19. Team RC. R: A Language and Environment for Statistical Computing. R Foundation for Statistical Computing, Vienna, Austria: https://wwwR-projectorg. 2015.
20. Dowle M, Short T, Lianoglou S. data. table: Extension of data. frame for fast indexing, fast ordered joins, fast assignment, fast grouping and list columns. R package version. 2013;1(8).
21. Gurobi Optimization I. Gurobi Optimizer Reference Manual. http://www.gurobi.com. 2015.
